# Biphasic and cardiomyocyte-specific IFIT activity protects cardiomyocytes from enteroviral infection

**DOI:** 10.1371/journal.ppat.1007674

**Published:** 2019-04-08

**Authors:** Taishi Kimura, Claudia T. Flynn, Mehrdad Alirezaei, Ganes C. Sen, J. Lindsay Whitton

**Affiliations:** 1 Department of Immunology and Microbiology, The Scripps Research Institute, La Jolla, California, United States of America; 2 Department of Inflammation and Immunity, Lerner Research Institute, Cleveland Clinic, Cleveland, Ohio, United States of America; University of Southern California, UNITED STATES

## Abstract

Viral myocarditis is a serious disease, commonly caused by type B coxsackieviruses (CVB). Here we show that innate immune protection against CVB3 myocarditis requires the IFIT (IFN-induced with tetratricopeptide) locus, which acts in a biphasic manner. Using IFIT locus knockout (IFITKO) cardiomyocytes we show that, in the absence of the IFIT locus, viral replication is dramatically increased, indicating that constitutive IFIT expression suppresses CVB replication in this cell type. IFNβ pre-treatment strongly suppresses CVB3 replication in wild type (wt) cardiomyocytes, but not in IFITKO cardiomyocytes, indicating that other interferon-stimulated genes (ISGs) cannot compensate for the loss of IFITs in this cell type. Thus, in isolated wt cardiomyocytes, the anti-CVB3 activity of IFITs is biphasic, being required for protection both before and after T1IFN signaling. These *in vitro* findings are replicated *in vivo*. Using novel IFITKO mice we demonstrate accelerated CVB3 replication in pancreas, liver and heart in the hours following infection. This early increase in virus load in IFITKO animals accelerates the induction of other ISGs in several tissues, enhancing virus clearance from some tissues, indicating that–in contrast to cardiomyocytes–other ISGs can offset the loss of IFITs from those cell types. In contrast, CVB3 persists in IFITKO hearts, and myocarditis occurs. Thus, cardiomyocytes have a specific, biphasic, and near-absolute requirement for IFITs to control CVB infection.

## Author summary

Viruses can infect the heart, causing inflammation–termed myocarditis–which is a serious, and sometimes fatal, disease. One way to combat the infection is by stimulating our immune system, encouraging it to fight the virus. However, the treatment that is currently used “revs up” many different parts of our immune system, including some that play little or no role in clearing the virus, and this wide-ranging activation increases the risk of potentially-harmful side effects. We want to identify the parts of the immune system that fight virus infections of the heart, so that we can improve the treatment of viral myocarditis by selectively stimulating only those immune responses, thereby retaining the benefit of treatment (i.e., clearing the virus) while reducing its cost (i.e. lowering the risk of harmful side effects). In this paper, we demonstrate that a family of proteins called IFITs play a role in protecting many tissues against these infections, but are particularly important in heart muscle cells, in which they are indispensable. Thus, IFITs represent a possible target for the treatment of viral myocarditis.

## Introduction

Myocarditis, which can cause serious, and sometimes fatal, complications including heart failure, cardiac arrest, and dilated cardiomyopathy, is commonly caused by infection and, most frequently, by viruses including coxsackievirus B3 (CVB3) [1, 2]. This enterovirus infects mice and humans, replicates to high titers, and causes acute viral myocarditis through two major pathological mechanisms; virus-mediated direct lysis of the infected cells and immune-mediated tissue damage (immunopathology). Limiting virus infection by activating the immune system through type I interferon therapy (T1IFN) has shown promise [3, 4], but comes with an increased risk of immunopathology, because T1IFNs have strong and pleiotropic biological effects. Therefore, it is important to better understand how T1IFNs exert their anti-enteroviral effects, with the aim of retaining their biological benefits while reducing concomitant immunopathology.

T1IFNs (mainly, ~12 subtypes of IFNα and the sole IFNβ in human and mouse) are important innate immune mediators against virus infection. T1IFN production is initiated in a virus-infected cell by the tripping of series of innate immune sensors; the resulting downstream signaling upregulates the transcription of genes encoding T1IFNs and pro-inflammatory cytokines, which are secreted from the infected cell [5]. After secretion, all of the T1IFN proteins signal through a common heterodimeric receptor, the T1IFN receptor (T1IFNR), expressed by the great majority of somatic cells. T1IFN binding to this receptor activates the JAK-STAT pathways, leading to the induction of a large number of interferon-stimulated genes (ISGs), which then exert various effects including innate immune antiviral action and modulation of cytokine production. Mice lacking this receptor rapidly succumb to CVB3 infection [6, 7], as do IFNβ knockout (KO) mice [8], demonstrating the essential role played by T1IFNs in protecting against this virus. We recently generated inducible conditional knockout mice (CM^MCM^ T1IFNR^f/f^ mice) in which the administration of tamoxifen efficiently deleted T1IFNR expression specifically in cardiomyocytes and, using these mice, we revealed the importance of local T1IFN signaling into cardiomyocytes during CVB3 infection. Without such signaling, at ~2–3 days post-infection (p.i.) we observed increased cardiac titers; myocarditis was accelerated, and virus clearance was delayed [7]. These data raised several questions: during CVB3 infection, which ISGs are induced in cardiomyocytes in response to CVB3 infection, which of these ISGs are needed to suppress virus replication, and which ISGs regulate the rapid influx of inflammatory cells into the heart?

We show here that, following CVB3 infection, IFIT (IFN-induced with tetratricopeptide) family genes are highly induced in cardiomyocytes *in vivo*. The IFITs are a large family comprising six murine (*Ifit1*, *Ifit2*, *Ifit3*, *Ifit1b*, *Ifit1c* and *Ifit3b*) and five human (*IFIT1*, *IFIT2*, *IFIT3*, *IFIT5 and IFIT1B*) members [9]. These genes exert antiviral responses against various different viral species by binding to both host and viral molecules [9–11], but the role of IFIT locus genes in enterovirus infection and the consequent pathogenesis have not been previously investigated. In this study, we use mice lacking the entire IFIT locus (IFITKO mice), several primary cell types from these mice, and cardiomyocytes modified by CRISPR/Cas9-mediated gene editing. Both of these approaches—*in vivo* and *in vitro*–indicate that the IFIT locus acts biphasically, and in a cardiomyocyte-specific manner. During the first phase, constitutive IFIT expression is required for suppressing early CVB3 replication in several tissues and cell types. In the second phase, which follows T1IFN signaling, the upregulation of IFITs is vital for CVB3 clearance from cardiomyocytes, and for the prevention of myocarditis. We conclude that the second phase of IFIT activity is cardiomyocyte-specific because the T1IFN-driven induction of IFITs is expendable in other cell types. In these cells–unlike in cardiomyocytes–other ISGs can provide compensatory anti-CVB3 activity, offsetting the absence of IFITs.

## Results

### Ifit1, 2, 3 and 3b are highly induced in cardiomyocytes during CVB3 infection

**Fig 1:**
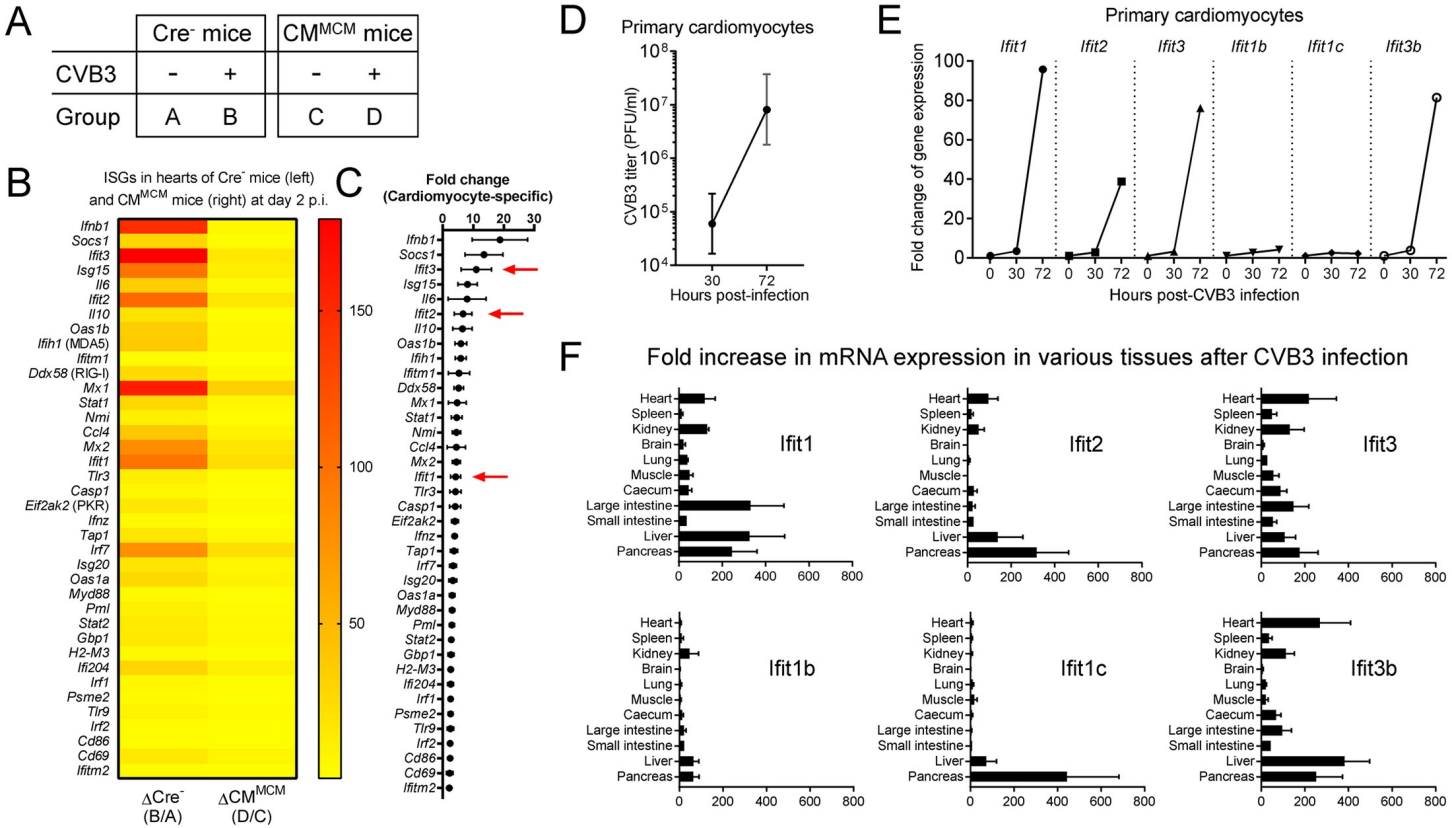
Ifit1, 2, 3 and 3b are highly induced in cardiomyocytes during CVB3 infection. Type I IFN-related gene expression was quantified by PCR array in the hearts of CVB3-infected (500 pfu i.p.) mice at 2 days p.i. (A) Schematic representation of the four different data sources subjected to PCR array analysis. (B and C) Only genes that are induced at statistically significant levels (*P* < 0.05) and more than 2-fold difference between Cre^-^ and CM^MCM^ mice are shown (n = 4–5, means ± SEM). (B) Heat map showing the fold-change of gene expression (compared to uninfected mice) after CVB3 infection in Cre^-^ mice (left column) and CM^MCM^ mice (right column). (C) Cardiomyocyte-specific changes in gene expression (left column of heatmap divided by right column of heatmap; IFIT genes are arrowed). (D and E) Primary cardiomyocytes were isolated from C57BL/6 (B6) mice and infected with CVB3 at MOI of 1. At indicated hours p.i., supernatants and cells were collected. (D) Infectious virus titers in the supernatant were determined by plaque assays and represented as PFU/ml. Data are representative of two independent experiments (n = 2, geometric means ± SD). (E) Real-time RT-PCR analysis of each IFIT family gene expression in primary cardiomyocytes. Each value was normalized to the values of *Gapdh* and divided by the values of uninfected controls (n = 1). (F) B6 mice were infected with 10^4^ PFU of CVB3 i.p., and IFIT mRNA expression was analyzed 48 hours later by real-time RT-PCR. Each value was normalized to the value of *Gapdh*, and divided by the values of uninfected controls (n = 3, means + SEM).

To identify which ISGs are expressed in normal cardiomyocytes during CVB3 infection *in vivo*, we exploited CM^MCM^ T1IFNR^f/f^ mice [7]. Two weeks after receiving a Tamoxifen injection, Cre^-^ or CM^MCM^ T1IFNR^f/f^ mice were challenged with 500 PFU CVB3 intraperitoneally (i.p.). Two days later, a time point when the cardiac virus titers are still comparable in both groups [7], the animals (and uninfected controls) were sacrificed, and ISG expression in the hearts of the four different data groups (Fig 1A) was determined by PCR array. CVB3 infection induced multiple ISGs in the genetically-intact heart (Fig 1B, left), but this was largely abolished by T1IFNR deficiency in cardiomyocytes (Fig 1B, right), indicating that ISG upregulation in the heart is limited mainly to those cells. By comparing the PCR signals of Cre^-^ and CM^MCM^ hearts, we estimated the extent to which various ISGs were upregulated in cardiomyocytes (Fig 1C). IFNβ mRNA was ~20-fold more abundant in the hearts of Cre^-^ mice, demonstrating that (i) cardiomyocytes are the major source of IFNβ during CVB3 infection and (ii) CVB3 infection triggers abundant IFNβ production by cardiomyocytes only if these cells can receive (or have already received) T1IFN signals. This is consistent with a previous report in which, using HL-1 cells (a murine cardiomyocyte cell line [12]), the authors showed that CVB3 infection did not directly trigger IFNβ production [13]. We have independently confirmed this finding (see S1 Fig). In addition to ISGs that been described previously (Ifnb1, Socs1, Isg15 and Il6) [8, 14–16], we found that several of the IFIT family genes were up-regulated in cardiomyocytes at 2 days p.i. (small arrows, Fig 1C; this PCR array assayed only Ifit1, Ifit2 and Ifit3). As described above, a broad spectrum of cellular functions of individual IFIT family genes, including antiviral effects, has been reported previously [9–11], but little is known about the collective importance of the IFIT locus, and less still about its role during enterovirus infection *in vivo*. Therefore, the remaining experiments reported herein focused on the role of this gene family in responding to CVB3 infection. To confirm the PCR array results *in vitro*, and to extend them to IFIT family members not covered in the PCR array, we isolated primary cardiomyocytes from C57BL/6 (B6) mice. CVB3 could efficiently infect, and replicate in, this cell type (Fig 1D). Real-time PCR analysis of CVB3-infected cardiomyocytes showed that, in the absence of exogenous T1IFNs, there is a substantial (> 30 hour) delay in IFIT expression, but by 72 hours p.i. there is robust induction of Ifit1, Ifit2 and Ifit3, and also of one previously-uncharacterized family member, Ifit3b. However, there was little, if any, induction of Ifit1b and Ifit1c at 72 hours p.i. (Fig 1E). Thus, our *in vivo* and *in vitro* data indicate that, by 48–72 after CVB3 infection, 3–4 members of IFIT family genes (*Ifit1*, *Ifit2*, *Ifit3* and *Ifit3b*) are highly induced in cardiomyocytes.

Next, we infected B6 mice with CVB3 (10^4^ pfu, i.p.). The animals (and uninfected controls) were sacrificed at 2 days after CVB3 infection, and IFIT family gene expression in 11 different tissues was analyzed by real-time RT-PCR (Fig 1F). Similar to cardiomyocytes, at this time point after CVB3 infection, *Ifit1*, *Ifit2*, *Ifit3 and Ifit3b* were induced in most tissues. Up-regulation of the other two family members is more tissue-restricted: *Ifit1b* is induced almost exclusively in kidney, liver and pancreas, and *Ifit1c* in liver and pancreas. In uninfected B6 mice, expression of most of the IFIT family mRNAs was low but detectable in most tissues (S2A Fig). We also determined the constitutive expression of the mRNAs in three primary cell types isolated from B6 mice (cardiomyocytes, peritoneal macrophages and cardiac fibroblasts), and found that all were expressed in uninfected cells (S2B Fig). The expression pattern of IFIT mRNAs differed between the two cardiac-derived cell types, reflecting others’ findings that basal levels of ISG expression–including IFIT1 –can be detected in both cell types, and that the expression pattern is cell-type-specific [17]. The relative levels of IFIT1 expression differed between the cited study and our own data; we speculate that this might result from unidentified differences in culture conditions. In contrast to the marked induction of most IFIT mRNAs at 48–72 hours after CVB3 infection *in vivo* and *in vitro* (Fig 1B, 1C, 1E and 1F), barely any increase was observed in mouse hearts at 24 hours p.i. (S2C Fig), supporting our observation that there was little increase at 30 hours p.i. (Fig 1E). Taken together, these data indicate that CVB3 infection of cardiomyocytes does not, in itself, drive the rapid and abundant expression of ISGs (including IFITs and T1IFNs); rather, the induction of ISGs depends on the cardiomyocytes having been primed by T1IFN signaling. Basal levels of IFIT2 and IFIT3 proteins were detectable in the liver and heart of naïve B6 mice, and both were markedly up-regulated following the *in vivo* administration of recombinant IFNβ (S2D Fig, left panels). Low constitutive expression of IFIT2 and IFIT3 proteins also was detectable in primary cardiomyocytes, and was up-regulated after 24 hours of IFNβ treatment (S2D Fig, right panels). Thus, we conclude that: (i) IFITs are constitutively expressed in many tissues / cell types; (ii) the constitutive expression pattern of the various IFIT genes can vary among cell types; (iii) CVB3 infection causes a robust increase in expression of most of the IFIT family genes, but (iv) in cardiomyocytes, this takes at least 30 hours to occur, suggesting that the increase depends upon these cells having received signals by systemic T1IFNs which, perhaps originate from other cell types, e.g. dendritic cells *in vivo*.

### T1IFN signaling into cardiomyocytes inhibits CVB3 infection in vitro

**Fig 2:**
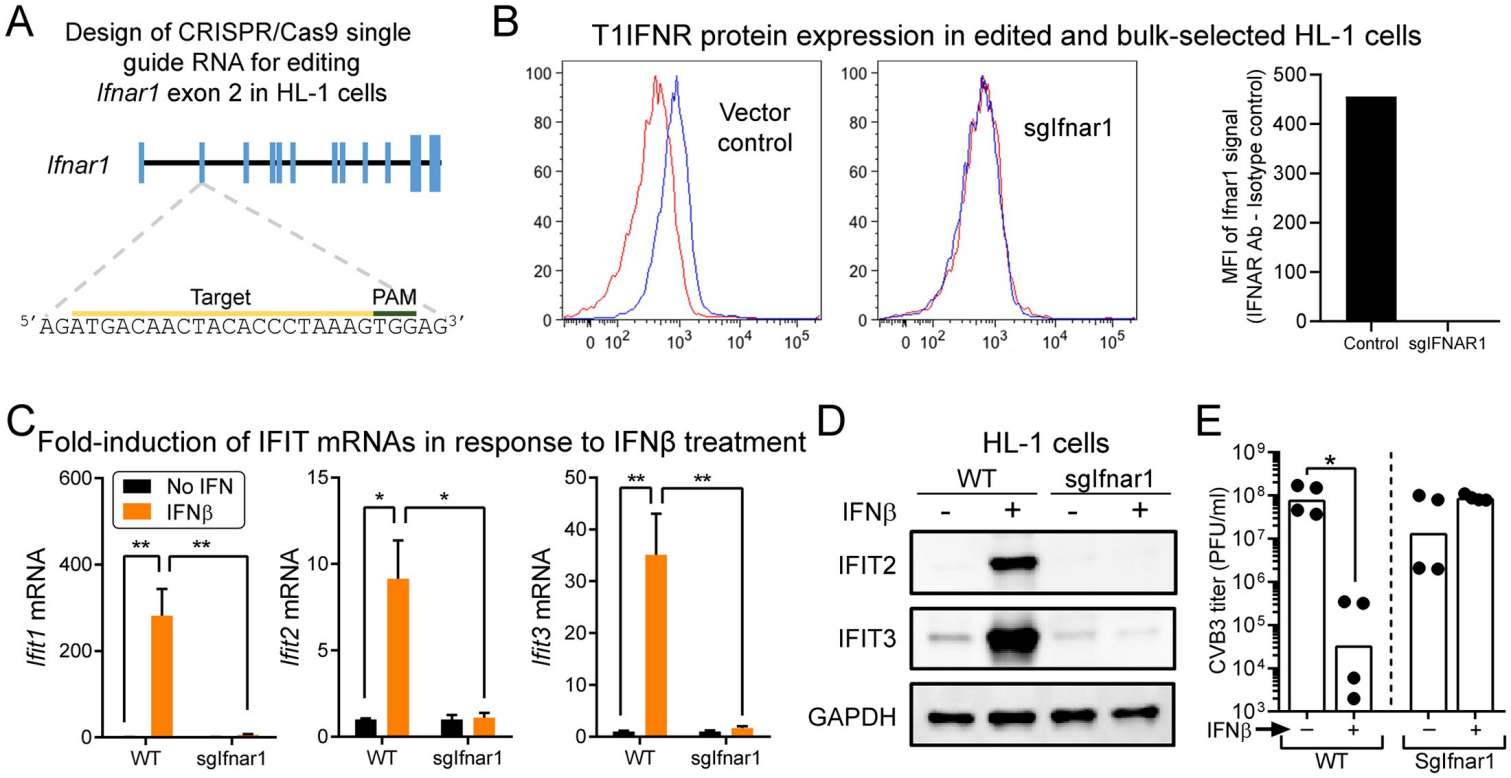
T1IFN signaling into cardiomyocytes inhibits CVB3 infection in vitro. (A-D) Generation of *Ifnar1*-edited (sgIfnar1) HL-1 cells, and characterization of uninfected cells. (A) Diagram of the *Ifnar1* gene locus relevant to this study. Closed box = exon. The location of the sgRNA target sequence for *Ifnar1* locus is shown. (B) IFNAR1 protein expression of HL-1 cells that were transfected with control vector, or vector encoding the sgRNA, was determined by flow cytometry (blue = IFNAR antibody; red = isotype control). Representative histograms are shown, along with a graph displaying the delta MFI for each population (Mean Fluorescent Intensity with IFNAR1 antibody—MFI with isotype control antibody). (C) Fold gene induction of *Ifit1*, *Ifit2* and *Ifit3* in WT and *Ifnar1*-edited HL-1 cells after 16 hours of IFNβ (100 U/ml) treatment. Each value was normalized to the values of *Gapdh* gene and divided by the values of untreated cells in each group (n = 3, Means + SEM). Note, y axes differ among the three graphs. (D) Western blots of the WT and *Ifnar1*-edited HL-1 cells showing constitutive and IFNβ induced IFIT protein expression. GAPDH was detected as internal controls. (E) WT or *Ifnar1*-edited (sgIfnar1) HL-1 cells were incubated with or without recombinant IFNβ (100 U/ml). 16 hours later, cells were infected with CVB3 at an MOI of 1. 72 hours p.i., infectious virus titers in the supernatant were determined by plaque assays. Data are combined from two independent experiments (n = 4, geometric means). Each symbol represents an individual value.

To study the antiviral effect of T1IFNs on CVB3 infection *in vitro*, we first employed HL-1 cells, which we modified using the CRISPR/Cas9 system [18]. To validate the approach, we began by transfecting HL-1 cells with a vector encoding a single guide RNA targeted to exon 2 of the *Ifnar1* gene, which encodes one of the heterodimeric T1IFN receptor proteins (Fig 2A). HL-1 cells did not expand after selection / single cell dilution, preventing us from developing HL-1 clonal lines. Therefore, we relied on bulk edited and selected HL-1 cells, in which T1IFNR protein expression was dramatically decreased (Fig 2B). Effective functional depletion was demonstrated by treating these cells, or their wt counterparts, with IFNβ; *Ifit1*, *Ifit2* and *Ifit3* mRNA induction was ablated in *Ifnar1*-edited HL-1 cells (Fig 2C). Western blotting showed that IFIT proteins were constitutively expressed at similar levels in wt and *Ifnar1*-edited HL-1 cells, but that IFNβ-driven induction of IFIT family gene products was ablated in the latter (Fig 2D). Finally, exogenous IFNβ pre-treatment reduced the production of infectious CVB3 by ~2,300-fold in WT HL-1 cells but had no suppressive effect in *Ifnar1*-edited HL-1 cells (Fig 2E). A further conclusion can be drawn from the data in this panel. There is no statistically-significant difference in virus titers between the non-treated WT and *Ifnar-1*-edited HL-1 cells, indicating that, during the time of infection, the cells did not produce sufficient IFNβ to confer any antiviral effect. This provides additional evidence that CVB3 infection of resting cardiomyocytes does not directly trigger abundant IFNβ production; if it did so, then one would have predicted that this endogenously-synthesized IFNβ would have suppressed viral replication in the WT cells to a level below that observed in *Ifnar-1*-edited cells, which are unable to respond to the cytokine. These findings confirm the *in vitro* data in S1 Fig, as well as our *in vivo* observations made using the CM^MCM^ T1IFNR^f/f^ mice; (i) T1IFN signaling into cardiomyocytes markedly inhibits CVB3 infection [7] and (ii) unless they receive T1IFN signals, cardiomyocytes do not produce abundant T1IFNs following infection by CVB3 (Fig 1). When combined with our demonstration that T1IFNs drive the strong up-regulation of IFIT expression in cardiomyocytes, these data raise the question: do IFIT genes participate in the observed T1IFN-mediated protection of cardiomyocytes against CVB3?

### The IFIT locus is required for anti-CVB3 innate immune response in cardiomyocytes in vitro

**Fig 3:**
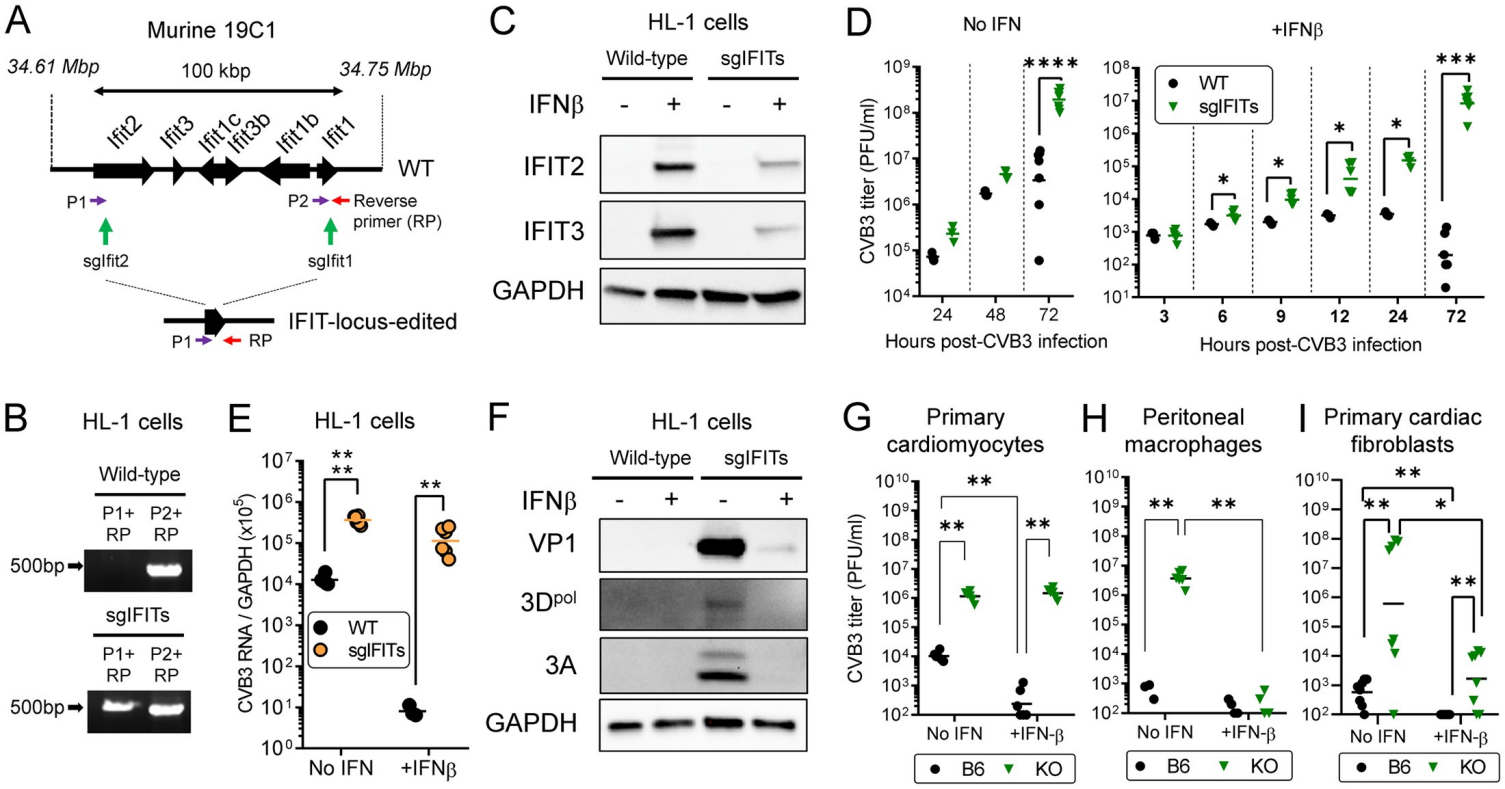
The IFIT locus is required for anti-CVB3 innate immune response in cardiomyocytes in vitro. (A-C) Generation of IFIT locus-edited (sgIFITs) HL-1 cells, and characterization of uninfected cells. (A) Black arrows = IFIT family genes. The locations of the PCR three primers (P1, P2 and Reverse) and sgRNA target sites spanning the IFIT locus are shown. (B) PCR results for the wt and bulk gene edited HL-1 cells are shown. (C) Western blots of the WT and IFIT locus-edited HL-1 cells. Some cells were treated for 16 hours with recombinant IFNβ (100 U/ml) to induce IFIT family proteins. GAPDH was detected as internal controls. (D-F) Phenotypical analysis of infected HL-1 cells. WT or IFITs-edited (sgIFITs) HL-1 cells were pre-treated with or without recombinant IFNβ (100 U/ml). 24 hours later, cells were infected with CVB3 at an MOI of 1. 72 hours p.i., cells and the supernatants were collected and used for subsequent studies. (D) Infectious virus titers in the supernatant were determined by plaque assays and represented as PFU/ml. Data are combined from three independent experiments (n = 3–10, geometric means). Each symbol represents an individual value. (E) Virus RNA accumulations in the cells at 72 hours p.i. were determined by real-time RT-PCR analysis. Each value was normalized to the values of *Gapdh*. Data are combined from two independent experiments (n = 6, geometric means). Each symbol represents an individual value. (F) Western blots of virus proteins (VP1, 3D^pol^ and 3A). GAPDH was detected as internal controls. (G-I) Virus titers in primary cells. Primary cardiomyocytes (G), peritoneal macrophages (H) and primary cardiac fibroblasts (I) were isolated from B6 and IFITKO mice and infected with CVB3 at MOI of 1. Then, some cells were treated with recombinant IFNβ (100 U/ml). 72 hours p.i., virus titers in the supernatants were determined by plaque assays. Data are combined from two independent experiments (n = 6–8, geometric means). Each symbol represents an individual value.

To address this question, we again applied the CRISPR/Cas9-mediated bulk gene-editing approach to HL-1 cells, this time deleting the entire IFIT locus. HL-1 cells were transfected with two CRISPR/Cas9 expression vectors expressing the indicated sgRNAs (Fig 3A) and, after drug selection, we confirmed the deletion of entire IFIT locus on genomic DNA of IFIT locus-edited HL-1 cells by PCR analysis (Fig 3B); the combination of primer P1 + reverse primer did not generate a detectable PCR product on WT DNA, because the two primers are separated by ~100 kbp, but a ~500 bp amplicon was present when DNA from the sgIFITs edited cells was used, indicating that these cells contained IFIT locus KO DNA. However, the bulk-edited and selected population was not 100% pure, because PCR using the reverse primer together with the P2 primer (which is absent from the KO DNA) produced an amplicon. To determine the impact of IFIT locus editing on IFIT protein content, IFIT2 and IFIT3 protein levels in HL-1 cells were determined by western blot without or with prior stimulation with recombinant IFNβ (100 U/ml). As shown in Fig 3C, robust induction of IFIT2 and IFIT3 was observed in WT cells, and this was markedly reduced in the sgIFIT population, although some protein was detected, consistent with the conclusion that the population contains some cells with intact IFIT genes. We then infected both populations of HL-1 cells with CVB3 (multiplicity of infection (MOI) of 1) in the presence or absence of IFNβ pre-treatment (100 U/ml). 72 hours p.i., virus titers in the supernatant of these cells were determined by plaque assays (Fig 3D). When cells were not pre-treated with IFNβ, infectious virus yield in the supernatant of IFIT locus-edited HL-1 cells was higher than in WT HL-1 cells at all time points, and became ~70-fold higher at 72 hours p.i. (p<0.0001) (Fig 3D, left panel). As shown in Fig 3D, right panel, IFNβ pre-treatment of WT HL-1 cells very effectively suppressed the infectious virus yield throughout the 72 hour time course (black symbols), whereas the virus still replicated to high titer in IFNβ-treated IFIT locus-edited HL-1 cells, in which the virus titer was significantly higher as early as 6 hours p.i., and ultimately became ~40,000-fold higher than in IFNβ pre-treated WT HL-1 cells (p = 0.0008). This viral titer data was reflected in analyses of virus RNA (Fig 3E). In the absence of IFNβ treatment, we found ~30-fold higher quantities of virus RNA in IFIT locus-edited HL-1 cells compared to WT cells (p<0.0001), and IFNβ pre-treatment reduced virus RNA in both cell populations, but much more so in the WT (*P* < 0.01). Parallel findings came from analyses of virus protein accumulation (Fig 3F). In summary, these data indicate that control of CVB3 infection by IFITs in HL-1 cardiomyocytes is biphasic: in the first phase, the constitutive (T1IFN-independent) expression of IFITs helps to constrain viral replication and in the second phase, T1IFN-driven up-regulation of IFIT expression in cardiomyocytes maintains this protection. The minimal antiviral effect of IFNβ on IFIT locus-edited cells (Fig 3D & 3E) indicates that, in cardiomyocytes, other ISGs are unable to confer substantial protection against CVB3.

To corroborate our HL-1 cell findings, we isolated primary cardiomyocytes from B6 mice and from IFIT locus-deleted (IFITKO) mice, which were generated in the laboratory of one of the authors (GCS) and will be described in detail in a separate paper. These IFITKO mice lack all of the IFIT family genes (*Ifit1*, *Ifit2*, *Ifit3*, *Ifit1b*, *Ifit1c* and *Ifit3b*), and the successful deletion is demonstrated by the fact that cardiomyocytes isolated from these IFITKO mice did not express any of the IFIT family genes, even after recombinant IFNβ treatment (S3 Fig). Primary cardiomyocytes from B6 and IFITKO mice were infected with CVB3, then recombinant IFNβ was (or was not) added to the culture media. 72 hours p.i., virus titers in the supernatant of these cells were determined (Fig 3G). These data confirmed the biphasic activities of IFITs in cardiomyocytes. CVB3 titer in the supernatant of primary cardiomyocytes (not treated with IFNβ) was significantly higher in IFITKO than B6 mice, and IFNβ treatment suppressed infection in WT cells, but not in IFITKO cells. Thus, in primary cardiomyocytes as in HL-1 cells, (i) IFIT locus genes appear to constitutively suppress CVB3 replication, (ii) IFN-driven IFIT up-regulation is required to maintain and extend this protection, and (iii) other ISGs, induced by exogenous IFNβ, appear unable to exert independent antiviral effects. We executed similar experiments with peritoneal macrophages, and with cardiac fibroblasts, from B6 and IFITKO mice (Fig 3H & 3I respectively). In both cell types, in the absence of exogenous IFNβ, virus yield was significantly increased in the IFITKO cells, similar to what was observed for cardiomyocytes. However, in contrast to cardiomyocytes, IFNβ treatment strongly inhibited infectious virus production by IFITKO macrophages and cardiac fibroblasts, although a stronger effect was observed for the former cell type. These data indicate that: (i) constitutive IFIT expression plays a role in suppressing CVB3 replication in cardiomyocytes, cardiac fibroblasts and peritoneal macrophages, and (ii) the second, T1IFN-induced, phase of IFIT activity shows cell specificity; if IFITs are absent, other ISGs can confer antiviral protection in macrophages, but not in cardiomyocytes, with cardiac fibroblasts showing an intermediate phenotype. Sherry and colleagues have previously proposed that cardiomyocytes, being non-replenishable cells, may have developed near-unique mechanisms to cope with viral challenges [19], and our observations support and extend this suggestion. Studies are ongoing to identify the precise mechanism(s) by which the IFIT locus mediates its anti-CVB3 activity.

### The IFIT locus limits early virus replication, contributes to subsequent mortality, and is required for a successful response to IFNβ

**Fig 4:**
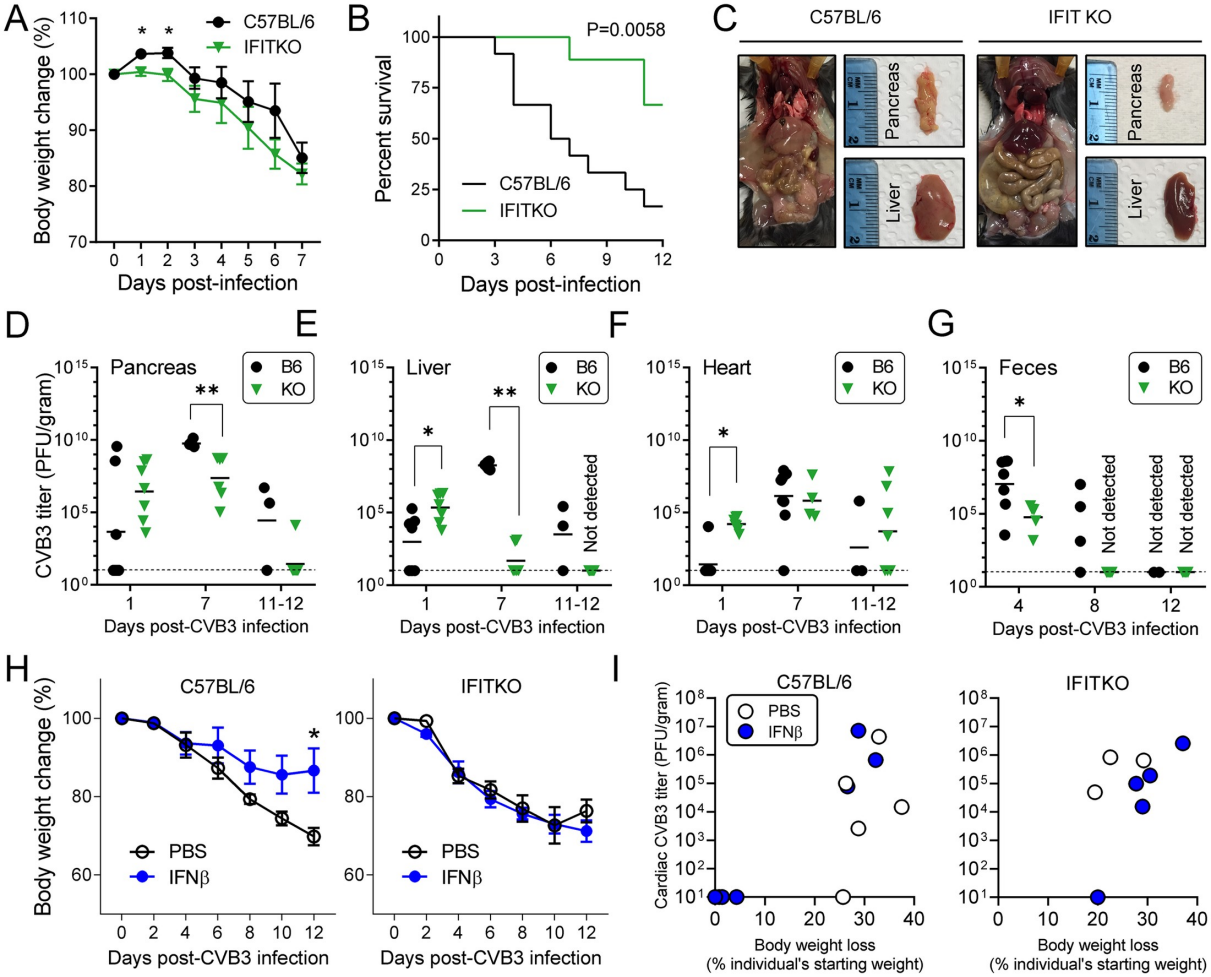
The IFIT locus limits early virus replication, contributes to subsequent mortality, and is required for a successful response to IFNβ. (A-G; black = B6; green = IFITKO mice) B6 and IFITKO mice were infected with CVB3 (10^4^ pfu/mouse, i.p.). (A) Body weight changes were monitored over a 7-day period. Body weights at the time of infection of each individual are set as 100% (n = 6, Means ± SEM). (B) Survival of both B6 (n = 12) and KO (n = 9) mice was assessed over a 12-day period. (C) Mice were sacrificed at 12 days p.i. and images of internal organs and extracted pancreata and livers were taken. (D-F) Mice were sacrificed at day 1 (n = 7 each), day 7 (B6, n = 7; KO, n = 6) and days 11–12 (B6, n = 3; KO, n = 7) p.i. Virus titers in the pancreas (D), liver (E) and heart (F) are represented as PFU/gram. The lower limit of virus detection was 10 PFU per gram of tissue sample (shown as dashed line). Each symbol represents an individual value. (G) Fecal samples were collected and virus titers were determined. (H & I: open circles = PBS-treated; blue circles = IFNβ treated). B6 and IFITKO mice were infected with CVB3 (10^4^ pfu/mouse, i.p.). 24 hours later, mice received either PBS or recombinant IFNβ. (H) Body weight changes were monitored over a 12-day period. Body weights at the time of infection of each individual were set as 100%. (I) The mice were sacrificed at 12 days p.i., and viral titers were determined in the heart (above) and in pancreata and livers (S6 Fig). The XY plots show, for the two mouse strains, the relationships among titer, body weight loss, and treatment status (PBS or IFNβ).

Next, we investigated the *in vivo* consequence of the loss of the IFIT locus during CVB3 infection. B6 and IFITKO mice were challenged with 10^4^ pfu of CVB3, i.p., and their body weights were monitored daily (Fig 4A). An early, and statistically-significant, loss of body weight was observed in IFITKO mice. Nevertheless, IFITKO mice survived significantly longer than B6 mice (Fig 4B). The CVB3-infected animals were sacrificed at 12 days p.i., and the two strains displayed dramatic macroscopic differences in the small intestine, pancreas, and liver (Fig 4C). The small intestine of CVB3-infected IFITKO mice was swollen and fulfilled with gas, and their pancreata were smaller than those of their B6 counterparts. In contrast, the livers of the IFITKO mice looked grossly normal, while those of B6 mice were pale, and confocal analyses revealed numerous apoptotic hepatocytes (S4 Fig), possibly contributing to the higher mortality in the WT animals (Fig 4B). This is consistent with the recent demonstration, by others, that hepatic disease may contribute significantly to the mortality associated with CVB3 infection [20]; we speculate that, in WT mice, the antiviral activity of IFITs in the liver may contribute to hepatic immunopathology that is detrimental to survival. No obvious macroscopic differences were observed in the hearts, but histological analyses were revealing (see below).

To analyze the *in vivo* impact of loss of the IFIT locus on CVB3 replication, we measured the virus titer in the pancreas, liver and heart of both groups at different time points over the course of CVB3 infection. At day 1 p.i., there were higher amounts of CVB3 in IFITKO mouse pancreas, liver and heart (Fig 4D to 4F), indicating that–as we showed for several IFITKO cell types *in vitro* (Fig 3G–3I)–IFIT locus genes are required for restricting very early CVB3 infection *in vivo*. However, at later time points (days 7, & 11–12 p.i.) CVB3 appeared to be more rapidly cleared from the pancreata and livers of IFITKO mice; at day 7 p.i., titers were ~300-fold lower in the IFITKO pancreata, and ~2 million-fold lower in the IFITKO livers (Fig 4D & 4E). We also found accelerated virus clearance in the feces of IFITKO mice (Fig 4G). These data indicate that, while the IFIT locus is required for immediate virus control in several tissues *in vivo*, its absence eventually leads to much more effective viral clearance from most of those tissues. The heart appears to be an exception: at day 7 p.i., CVB3 titers were high in the hearts of both mouse strains the small difference observed at this time point (~2-fold higher in the B6 mice) was far less than the differences observed in pancreata and livers and, by day 11–12 p.i., titers in IFITKO pancreata and liver were far below those in WT tissues (Fig 4D & 4E), while the titers in IFITKO hearts were ~20-fold higher than those in B6 mice (Fig 4F). This conclusion was supported by studies in which mice were challenged with a lower dose of CVB3 (10^3^ pfu); IFITKO mice showed 100-fold higher cardiac titers compared to B6 mice (*P* < 0.05; S5A Fig).

Exogenous IFNβ treatment has been shown to improve CVB3-induced pathogenesis in mouse models [21, 22] and in some human clinical trials [3, 4]. Since our *in vitro* data (Fig 3D & 3E) had shown us that the IFIT locus was required for most of the T1IFN-mediated inhibition of CVB3 replication in cardiomyocytes, we next examined whether the IFIT locus is required for IFNβ-mediated beneficial effects *in vivo*. B6 and IFITKO mice were challenged with 10^4^ PFU of CVB3 and, 24 hours p.i., received a single i.p. injection of either PBS or recombinant IFNβ (2 × 10^4^ units). We monitored the body weight loss of these animals, and found that IFNβ treatment protected B6 mice from weight loss and overt signs of disease over a 12 day period of CVB3 infection, but failed to do so in IFITKO mice (Fig 4H). Surviving mice were sacrificed at day 12 p.i., and virus titers were measured in the pancreas, liver, and heart. For the heart, data are shown in Fig 4I; data for pancreata and livers are shown in S6 Fig. Three conclusions may be drawn: (i) in both mouse strains, there was a clear relationship between substantial body weight loss and high cardiac titers; (ii) most of the B6 mice benefited from IFNβ (cleared virus from the heart, and showed minimal weight loss); and (iii) most importantly, for the purpose of our study, no such effects were observed in IFITKO mice (only one of the IFITKO mice had cleared virus from the heart, and that mouse still had significant weight loss). These data are consistent with the near-complete requirement for IFITs in cardiac clearance. Similar conclusions can be drawn from the pancreatic data, but for the liver it is more difficult to interpret any relationships with virus titer, because the great majority of the livers in both strains scored negative at this time point p.i. (S6 Fig).

### ISG expression is generally increased in IFITKO mice at 48 hours after CVB3 infection

**Fig 5:**
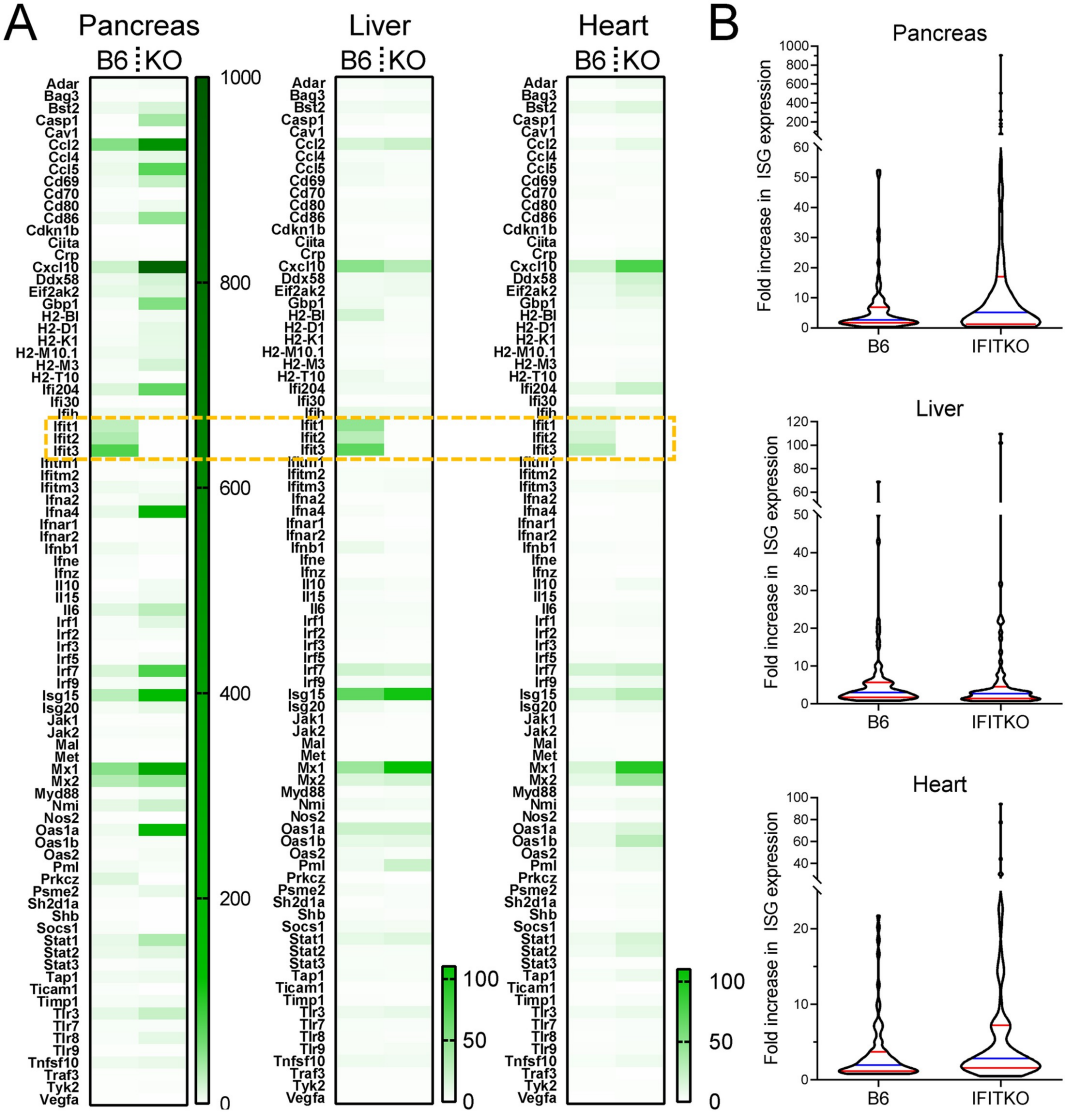
ISG expression is generally increased in IFITKO mice at 48 hours after CVB3 infection. B6 and IFITKO mice (n = 3 per group) were infected with 10^4^ pfu of CVB3, and were sacrificed 48 hours later. Pancreata, livers and hearts were harvested, RNA was extracted and the expression of 84 ISGs was analyzed using a mouse T1IFN response profiler PCR array. RNAs from organs of uninfected mice were used as controls. (A) Heat maps showing the fold gene induction (compared to uninfected tissues) in the three organs from infected B6 and IFITKO mice. The IFIT genes (negative in the heat maps for the IFITKO mice) are enclosed in a dashed gold box. (B) To more easily visualize the overall expression patterns in the B6 and IFITKO organs, the same data are graphed using a violin plot. Blue line = median; red lines = 25th and 75th quartiles. Because they are, by definition, absent from IFITKO mice, the IFIT genes were excluded from these violin plots; thus, these plots compare the expression of 81 ISGs in B6 and IFITKO mice.

Next, we sought to determine why virus clearance is accelerated in pancreas and liver, but not in heart, of IFITKO mice at later time points (days 7 & 11–12 p.i.). Since viral load at early time points is higher in IFITKO cells (Fig 3D) and in IFITKO mice (Fig 4D–4F), we reasoned that this might lead to the more rapid induction of innate responses in these mice compared to B6 animals. Strikingly, at day 1 p.i., several chemokine mRNAs were highly induced in the hearts of IFITKO mice, but only modestly so in B6 tissues (S7 Fig). We chose to carry out a more detailed analysis at 48 hours p.i., because we knew that, by this time point, systemic T1IFNs have driven the upregulation of many ISGs in the infected heart (see Fig 1B & 1C). PCR array heat maps (Fig 5A) revealed that, at 48 hour p.i. many ISGs appeared to be expressed at higher levels in IFITKO tissues than in their WT counterparts. Of interest, too, the pattern of ISG induction differed among the three organs analyzed. To more readily visualize the overall differences between the two mouse strains, the data were re-plotted to compare the range and extent of ISG up-regulation in all three organs of both strains (Fig 5B). In all three organs, the highest ISG up-regulation occurred in IFITKO conditions, and the overall range of ISG expression appeared higher in IFITKO pancreas and heart, and almost equivalent in livers from both mouse strains. Thus, we consider it likely that this increased ISG expression may underpin the faster resolution of infection in the pancreas and liver of IFITKO mice (Fig 4D & 4E). This enhanced virus clearance does not occur in the IFITKO heart (Fig 4F) despite there being increased overall ISG expression (Fig 5B), consistent with the notion that, at least in cardiomyocytes, other ISGs cannot functionally compensate for the loss of the IFIT locus.

Taken together, these *in vivo* data indicate that constitutive IFIT expression plays a key role in restricting CVB3 replication in most/all tissues and, in its absence, virus RNA-driven induction of the T1IFN response is accelerated; this, in turn, leads to the rapid up-regulation of a variety of other ISGs in all tissues, and these ISGs quickly control CVB3 replication in all of the tested tissues, except the heart. Thus, we suggest that the IFIT locus is especially vital for protecting cardiomyocytes, because these cells lack the functional redundancy that the other ISGs can provide in most cell types.

### The IFIT locus is required for preventing CVB3-induced myocarditis

**Fig 6:**
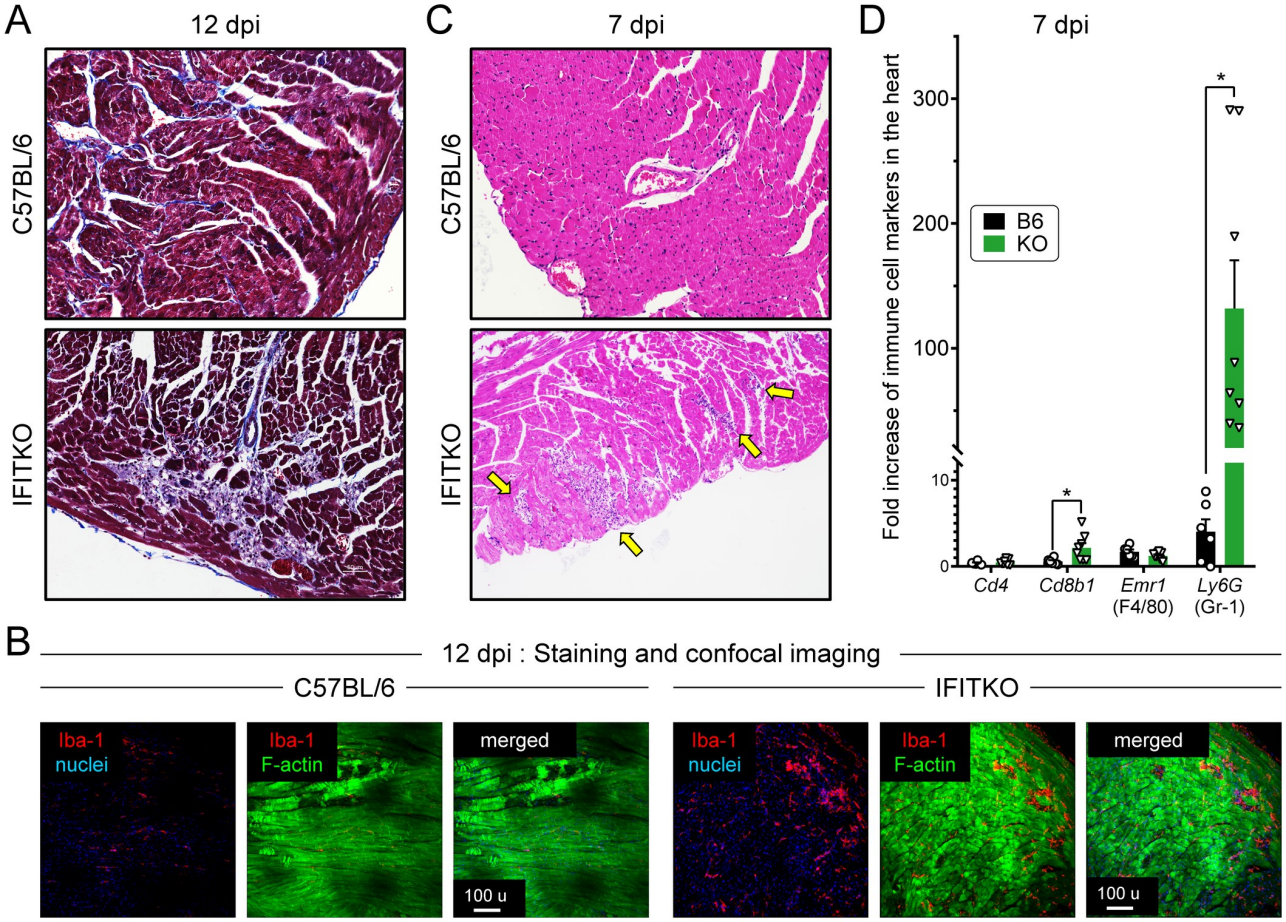
The IFIT locus is required for preventing CVB3-induced myocarditis. C57BL/6 (B6) and IFITKO (KO) mice were infected with CVB3 (10^4^ pfu/mouse, i.p.). (A) Representative histological sections of hearts stained with Masson’s trichrome (12 days p.i.) are shown. (B) Vibratome sections of the above hearts were stained as indicated, and were imaged by confocal microscopy. Iba-1 (macrophages) (Red), F-actin (Green), and nuclei (Blue). (C) Representative hematoxylin and eosin (H&E) -stained sections of hearts stained taken at 7 days p.i. are shown. Yellow arrowheads indicate sites of cellular infiltration. (D) The extent of this infiltration, and its cellular composition, was assessed using RT-PCR for the four indicated RNAs: Cd4, Cd8b1, Emr1 (F4/80), Ly6G (Gr-1). (B6, n = 6; KO, n = 8, Means + SEM). Each symbol represents an individual value.

Finally, we investigated the impact of loss of the IFIT locus on the extent of CVB3-induced myocarditis. Mice were infected with 10^4^ pfu CVB3 i.p. and, 12 days later, hearts were harvested, and paraffin sections were stained (Fig 6A). Immune cell infiltration was quite limited in the B6 heart at 12 days p.i., while numerous infiltrating cells, and collagen deposition (light blue), were observed in the hearts of IFITKO mice. These findings were reproducible when mice were challenged at a dose of 10^3^ pfu of CVB3 (S5B Fig). Since we had observed enhanced chemokine expression in the IFITKO hearts soon after infection (S7 Fig), we analyzed infiltration of macrophages by staining heart vibratome sections with an antibody against Iba-1 (Ionized calcium-binding adaptor molecule 1, also known as Aif-1), a protein that is predominantly expressed on cells of the macrophage lineage [23]. At 12 days post-CVB3 infection (10^4^ pfu), Iba-1 signals (Fig 6B, red) were brighter and more frequent in the hearts of CVB3-infected IFITKO mice compared to the WT animals. Our previous work showed that development of myocarditis in the mice lacking T1IFN signaling into cardiomyocytes was not only exacerbated but also accelerated [7]. Likewise, we found more myocardial inflammation in IFITKO than in B6 mice at 7 days p.i. (Fig 6C, yellow arrows indicate immune infiltrates), a time point when the mice of both groups showed comparably high cardiac virus titers (Fig 4F). Real-time RT-PCR analysis was applied to RNA extracted from the hearts of both mouse strains at d7 p.i., to identify genes expressed by immune cells, and revealed a small but significant increase of *Cd8* RNA, and a massive increase of Ly6G, a marker of granulocytes and monocytes, in IFITKO hearts (Fig 6D). Therefore, in the absence of the IFIT locus, there is more rapid inflammatory cell infiltration into the heart. Taken together, these data indicate that the IFIT locus plays an important role in limiting CVB3-induced myocarditis.

## Discussion

The present study was aimed at identifying the genes responsible for T1IFN-dependent antiviral protection in the heart. We report: (i) that the IFIT locus plays a central role in controlling CVB3 infection in multiple tissues including the heart; (ii) that it does so in two distinct phases, separated by the onset of T1IFN signaling; (iii) that the first phase, which depends on constitutive IFIT activity, impacts all analyzed tissues; but (iv) that the second, T1IFN-induced, phase of IFIT activity is cell-specific, being almost indispensable in cardiomyocytes, and redundant in other cell types.

Most cell types can respond to T1IFNs, thereby increasing their ability to resist virus challenge. However, prior to being stimulated by T1IFNs, many cells also have a constitutive capacity to withstand virus infection. This has been referred to as “intrinsic antiviral immunity” [24], and here we demonstrate that the IFIT family genes play such a role in protecting many cell types and tissues against CVB3 infection. Our *in vitro* observations show that, in the absence of T1IFN treatment, CVB3 replicates to higher titers in IFIT-deficient cardiomyocytes (both in HL-1 cells and in primary isolates), peritoneal macrophages, and cardiac fibroblasts (Fig 3G–3I). Others have reported that, specifically in cardiomyocytes, the mitochondrial antiviral signaling (MAVS) pathway is spontaneously activated, resulting in increased basal levels of IFNβ [25], suggesting the possibility that IFNβ might contribute to the intrinsic immunity of cardiomyocytes to CVB3 that we report herein. However, as noted above, CVB3 infection of primary cardiomyocytes does not trigger abundant IFNβ production. Furthermore, it is intriguing to note that some viral proteases have been shown to cleave MAVS protein, potentially nullifying the pathway’s activity; these include the 3C protease of CVB3 [26], and the 3ABC complex of another picornavirus, hepatitis A virus [27]. Moreover, cytokine responses to CVB3 appear to be independent of MAVS, and CVB3 titers are not increased in MAVS-deficient mice [28]. Given that, both before and after IFNβ stimulation, cardiomyocytes display a near-absolute requirement for endogenous IFITs (Fig 3D–3G), we consider it likely that one or more of the proteins in the IFIT family play(s) the key role in conferring both constitutive and inducible anti-CVB3 protection in these cells.

The importance of constitutive IFIT expression was confirmed by *in vivo* studies. IFITs are constitutively expressed in many tissues and cell types (S2 Fig panels A, B and D, and Fig 2D) and, compared to WT mice, CVB3 titers at 1 day p.i. were markedly higher in multiple tissues of IFITKO mice (Fig 4D–4F). By two days after CVB3 infection, mice have transitioned from the first phase of antiviral immunity (cell-intrinsic resistance) to the second, T1IFN-induced, phase. At this time point, genetically-intact hearts express many ISGs (Fig 1B & 1C), one of which is IFNβ, whose abundance is reduced ~20-fold if cardiomyocytes are unable to respond to T1IFN (Fig 1C). These data suggest that cardiomyocytes are, by far, the major source of IFNβ in the CVB3-infected heart. Moreover, they demonstrate that extensive T1IFN synthesis by cardiomyocytes requires that the cells be able to respond to the cytokine–i.e., wt cardiomyocytes exhibit a positive feedback loop *in vivo*, leading to the escalation of local T1IFN concentration, with consequent rapid and marked induction of numerous other ISGs, including several IFITs (Fig 1B and 1C). This is consistent with the *in vitro* and *in vivo* observations that suggest that there is a 1–2 day delay in IFIT up-regulation, followed by an explosive increase. What are the antiviral consequences of T1IFN signaling into cardiomyocytes? As noted above, we have reported that T1IFNR-deficient cardiomyocytes show delayed clearance of CVB3 *in vivo* [7], and here we confirm *in vitro* the importance of T1IFN signaling in cardiomyocytes; IFNβ treatment reduced the yield of infectious virus by ~2,300-fold in wt HL-1 cells, while no such effect was observed using CRISPR/Cas generated T1IFNR-deficient cardiomyocytes (Fig 2E).

Strikingly, this T1IFN-driven suppression of CVB3 infection in cardiomyocytes is almost entirely dependent on the IFIT locus. IFNβ-treatment of wt HL-1 cells or wt primary cardiomyocytes dramatically inhibited CVB3 replication, but equivalent treatment of IFIT-deficient cardiomyocytes had very little effect, suggesting a near-absolute requirement for the IFIT locus in this cell type (Fig 3D to 3G). In contrast, IFNβ-treated IFITKO peritoneal macrophages very efficiently controlled the infection, while IFNβ-treated cardiac fibroblasts showed an intermediate phenotype (Fig 3H & 3I) indicating that, for both of these cell types, other ISGs could wholly or partially restore antiviral resistance. Thus, IFITs act in a biphasic manner following CVB3 infection, and the T1IFN-driven second phase shows cell-type specificity. This mirrors previous reports of neuron-specific antiviral activity of IFIT2 against vesicular stomatitis virus [29, 30]. The biological importance of this T1IFN-driven shift, from intrinsic immunity (phase 1) to ISG-mediated antiviral responses (phase 2) at ~1–2 days p.i. is clearly shown by the prior observations that IFNβKO, T1IFNRKO and CM^MCM^T1IFNR^f/f^ mice all failed to control CVB3 infection [6–8], demonstrating that intrinsic immunity alone is insufficient. Furthermore, our data suggest that intrinsic immunity–or, at least, the intrinsic immunity conferred by the IFIT locus–can be lost without fatal effects. In IFITKO mice, virus titers were initially higher than in wt animals–perhaps explaining the early and transient weight loss that occurred in these mice (Fig 4A)–but at later time points virus clearance was accelerated in the pancreas, liver and feces (Fig 4D, 4E & 4G). This enhanced virus clearance is probably attributable to the more rapid induction of T1IFNs and ISGs in IFITKO mice (Fig 5), presumably driven by the extremely high viral titers that were present at 1 day p.i. Consistent with our *in vitro* data, this accelerated CVB3 clearance showed substantial cell (tissue) specificity: it was not observed in the hearts of IFITKO mice which, at 12 days p.i., still contained much higher levels of CVB3 than did WT hearts (Fig 4F & 4I). In addition, cardiac CVB3 titers in IFITKO mice were prolonged even if the mice were treated with exogenous IFNβ (Fig 4I). Taken together, these findings suggest that other ISGs can substitute for the absence of IFITs in many tissues, but not in the heart, because of cardiomyocytes’ requirement for IFITs. The inability of other ISGs to protect cardiomyocytes against CVB3 after ~day 2 p.i., when the T1IFN system has exerted its effects, appears to render these cells a more hospitable environment for the virus.

As mentioned in the Introduction, virus-mediated direct cell lysis and immunopathology are the two major pathological mechanisms of myocarditis. Previous studies in mice in which the viral receptor (CAR) has been deleted in cardiomyocytes found that the hearts of these mice are resistant to infection, and the mice are largely protected against cardiac disease [31, 32], demonstrating that virus replication in cardiomyocytes is a prerequisite for myocarditis. Our data showed that enhanced cardiac virus replication in IFITKO mice was accompanied by accelerated and exacerbated myocarditis (Fig 6 and S5B Fig). However, in contrast to our tissue culture data, which show clearly that IFIT expression in cardiomyocytes is the key factor, these *in vivo* data in IFITKO mice–which show that IFITs protect against myocarditis–are open to interpretation. It is possible that, in genetically-intact mice, protection against myocarditis is mediated solely by IFITs in cardiomyocytes (paralleling the tissue culture data), but it also is possible that protection is mediated, at least in part, by the early actions of constitutively-expressed IFITs in multiple tissues. This issue can be resolved in the future by generating mice carrying a floxed IFIT locus, and crossing them to CM^MCM^ mice [which are described in ref 7], allowing the inducible deletion of the locus specifically from cardiomyocytes. Whichever mechanism is in play, the primary means by which the IFIT locus prevents viral myocarditis in WT animals is to inhibit CVB3 replication, thereby reducing both virus-mediated direct cell lysis and the immunopathological damage caused by infiltrating cells. In addition, we observed that the increased early viral load in the IFITKO mice is accompanied by a more rapid T1IFN response (Fig 5). Interestingly, in Sendai virus (SeV) -infected *Ifit2* KO mice, which show elevated T1IFN induction triggered by uncontrolled virus replication, both SeV infection and abnormal production of T1IFN are required for the virus pathogenesis [33]. Hence, during CVB3 infection of IFITKO mice, the rapid / robust induction of the T1IFN response together with the prolonged virus presence in the heart may synergistically promote the cardiac immune cell infiltration and contribute to the pathogenesis of myocarditis.

In conclusion, we have revealed a key role for the IFIT locus in modulating CVB3 infection. Through analyzing genetically-manipulated cells and mice, we show that the IFIT locus constitutively limits early virus replication in many tissues, and that its subsequent upregulation by the T1IFN response plays an especially-important role in cardiomyocytes, delaying or preventing the development of myocarditis. Future studies to reveal the precise mechanism(s) by which the IFIT locus acts, and to determine the function of each IFIT family gene, may lead to new and improved strategies for treating enterovirus-induced disease.

## Materials & methods

### Ethics statement

All animal experiments were approved by The Scripps Research Institute (TSRI) Institutional Animal Care and Use Committee (protocol number 09-0131-3) and were carried out in accordance with the National Institutes of Health’s Guide for the Care and Use of Laboratory Animals.

### Mice

C57BL/6 mice were purchased from the TSRI rodent breeding colony. Generation of IFITKO mice by the Sen laboratory will be described in detail elsewhere. CM^MCM^ T1IFNR^f/f^ mice were described previously [7].

### Mouse serum samples

Mice serum was isolated using K3-EDTA coated microvette tubes (Starstedt, Nümbrecht, DEU) from the blood by centrifuging at 12,000 rpm for 15 min at room temperature, and stored at -20 °C until its use.

### Cells

The cardiomyocyte cell line HL-1 [12] was obtained from Drs. William C. Claycomb and Ikuo Tsunoda at Louisiana State University (Shreveport, USA). The cells were cultured in Claycomb medium supplemented with 100 μM norepinephrin, 10% fetal bovine serum (FBS) and 4 mM L-glutamine, in 37°C, 5% CO_2_ atmosphere. The Pierce primary cardiomyocytes isolation kit (Thermo Fisher Scientific, #88281) was used to isolate primary cardiomyocytes and primary cardiac fibroblasts, following the manufacturer’s instructions with some modifications [17]. In brief, day 2 post neonate mice were sacrificed and the hearts were isolated. After mincing each heart into 1–3 mm^3^ pieces, the tissues were washed twice with HBSS (Hanks-based salt solution), resuspended and incubated in working solution including primary cardiomyocyte isolation enzymes 1 and 2 (components of the kit) at 37°C for 30 minutes. The tissues were washed with HBSS several times and then with DMEM. Cells were plated at a density of 1.25 x 10^6^ per well in six-well plates for 2 hours in order to separate cardiac fibroblasts (rapidly adhering) from cardiomyocytes (still floating at 2 hours after plating). Adherent cells were washed with PBS and cultured for nine days, changing media every 3 days. Then, cell clusters that do not include any beating cells were collected and used as primary cardiac fibroblasts. For cardiomyocyte culture, 24 hours after plating the cells, fresh medium was added, with cardiomyocyte differentiation supplement (another component of the kit). After 7 days’ growth and differentiation with one medium change at day 4, cells were used as primary cardiomyocytes. For peritoneal macrophage isolation, mice were injected with aged 3% thioglycollate medium (SIGMA Aldrich, #T9032) i.p.. Three days later the mice were sacrificed, and peritoneal cells were recovered by lavage and seeded onto a tissue culture plate. The next day, floating cells were removed, and the adherent cells were used as macrophages.

### Viruses

The wild-type CVB3 used in these studies is a plaque purified isolate (designated H3) of the myocarditic Woodruff variant of CVB3. Mice were infected intraperitoneally (i.p) with the indicated dose of CVB3 and their survival and body weight were monitored during the course of infection. At the indicated times p.i., feces were collected, and at the time of sacrifice, pancreata, livers and hearts were isolated, weighed, and homogenized in 1 ml Dulbecco modified Eagle medium (DMEM). Virus titers were assessed using standard plaque assays as previously described [34].

### PCR array analysis and real-time RT-PCR

T1IFN-related gene expression in pancreata, livers and hearts was quantified using Mouse Type I interferon response RT2 Profiler PCR Array (PAMM-016Z, SA Biosciences, Frederick, MD). For real-time RT-PCR analysis, RNA was isolated from tissue and cell suspensions using the RNeasy Mini Kit (QIAGEN, # 74104), and 1–2 μg of RNA was reverse transcribed using iScript Reverse Transcription supermix (Bio-rad, #1708841). Real-time PCR was performed using Power SYBR Green PCR mastermix reagent (Applied biosystems, #4367659) with specific primer sets (see S1 Table). All of the values in PCR array analysis and real-time PCR analysis were normalized to the values of *Gapdh*.

### CRISPR-mediated gene editing

2 x 10^5^ HL-1 cells were seeded onto gelatin/fibronectin-coated plate. 24 hours later, pX459 (ver. 2) encoding either sgIfnar1 or sgIfit1 and sgIfit2 was transfected into HL-1 cells and incubated for further 24 hours. The sequences of the sgIFITs are shown in S1 Table. Then culture medium was changed to the media containing puromycin (3 μg/ml) and Cas9-expressing cells were selected for three days. After drug selection, puromycin was removed from culture media and cells were recovered. These bulk gene-edited cells were used for *in vitro* studies at early passage numbers.

### Flow cytometry

To determine surface IFNAR1 protein expression, HL-1 cells were analyzed by flow cytometry. WT or Ifnar1-edited HL-1 cells were incubated in trypsin-EDTA at 37°C for 5 min. Then, the reaction was stopped by adding DMEM supplemented with FBS. After washing several times, isolated cells were incubated with PE-conjugated anti-IFNAR1 antibody or PE-conjugated isotype control on ice for 20 min. After washing several times with FACS buffer, IFNAR1 expression on HL-1 cells was analyzed by flow cytometry using an LSR II (BD Bioscience).

### BCA assays and western blotting

Cells were lysed in RIPA buffer (Millipore, #20–188). After centrifugation, cell debris was discarded and protein concentration in the supernatant was determined by Pierce BCA Protein Assay Kit (Thermo Fisher Scientific, #23225). Colorimetry was measured using plate reader, Victor X3 (Perkin Elmer). 5 μg of total cell lysates were mixed with Laemmli Sample Buffer (Bio-Rad, #161–0747) and 10% of 2-Mercaptoethanol (SIGMA Aldrich, #M6250) and used for western blotting. Blotting of the proteins to membrane was performed using Trans-blot Turbo RTA Transfer Kit (Bio-rad, #170–4272) as follows. After developed on SDS gels, proteins were transferred on PVDF membranes (Bio-Rad, a component of the Transfer Kit) by using Trans-blot Turbo system (Bio-Rad). Membranes were then blocked with 1% skim milk for an hour, and overnight with the relevant diluted primary antibodies. Then, membranes were washed three times with Tris buffer Tween 20 (TBST) and incubated with diluted secondary antibodies. One hour later, membranes were washed again three times with TBST, then protein-antibody complexes were visualized by Super Signal ELISA Femto Maximum Sensitivity Substrate (Thermo Scientific, #37074).

### Histological analysis

Mice were perfused with Dulbecco’s PBS (DPBS), and tissues were harvested and fixed using buffered zinc formalin at room temperature (RT) overnight. For standard histological analyses, tissues were paraffin embedded, and 3-μm sections were cut and stained with hematoxylin-eosin or Masson’s trichrome. Images were captured at 10x magnification with an BZ-X710 inverted microscope (KEYENCE) using BZ-X Viewer software (KEYENCE). For confocal studies, 70 μm sections of liver and heart were cut with a Leica VT 1000S vibratome. Sections were incubated with primary antibody for 1 hr at RT and then at 4°C overnight. After washing, they then were incubated with secondary antibody for 1 hr at RT, washed and then incubated with Phalloidin 488 at 4°C overnight to label F-actin. After incubation, sections were washed, counterstained with Hoechst 33342 and mounted with ProLong Gold Antifade Mountant for confocal microscopy. Confocal images were captured using a Zeiss LSM 710 Laser Confocal Scanning Microscope running Zen 2009 Zeiss software suite. Representative regions within each vibratome section of the tissues were scanned as 8-bit optical sections (1,024 × 1,024 image sizes) and reconstructed for analysis. Exposure and image acquisition settings were identical for all sections.

### Statistical analyses

All data were analyzed using Prism software (GraphPad Prism 8). An unpaired, two-tailed t-test was used to determine statistically significant differences for *in vitro* experiments. The Mann-Whitney test was used to analyze differences in viral burden. Kaplan-Meier survival curves were analyzed by the log rank test. P values less than 0.05 were considered significant, and are indicated in figures as follows: * 0.05>p>0.01; ** 0.01≥p>0.001; *** 0.001≥p>0.0001; **** p≤0.0001.

## Supporting information

S1 Fig CVB3 infection induces abundant IFN-b transcription in cardiomyocytes only if the cells have been pre-treated with T1IFN (related to Fig 1). HL-1 cells were pre-treated (or not) with IFN-b for 16 hours. The cells were washed, and aliquots were infected with CVB3 at an moi of 10. 3 hours later, the cells were harvested and RNA was isolated. The abundance of IFN-b transcripts was determined using qPCR. As shown, (i) consistent with published data (see main text), CVB3 infection alone induces only a small, and statistically non-significant, increase in IFNβ in cardiomyocytes; (ii) IFNβ alone causes a statistically-significant increase in its own transcription and (iii) in IFNβ-pretreated cells, CVB3 infection now results in a dramatic (25-fold) increase in IFNβ transcript levels compared to untreated, non-infected cells. Taken together with our in vivo observations (Fig 1B & 1C), these data indicate that both in vivo and in tissue culture, the abundant transcription of IFNβ requires both T1IFN signaling and CVB3 infection.
(TIF)

S2 Fig Constitutive (A & B), virus-induced (C) and IFN-inducible (D) expression patterns of IFIT family genes in multiple tissues and primary cell lines (related to Fig 1). (A) Uninfected B6 mice were sacrificed and RNA was isolated from the indicated tissues. (B) Primary cardiomyocytes, peritoneal macrophages and cardiac fibroblasts were obtained from B6 mice, and RNA was isolated. For A & B, expression of mRNAs from the indicated IFIT family genes were analyzed; each value was normalized to the value for the Gapdh mRNA. (C) B6 mice were infected with CVB3 (10^4^ pfu/mouse, i.p.) and, 24 hours later, were sacrificed. Fold changes in the expression of IFIT mRNAs (compared to uninfected hearts) were determined. (D) B6 mice (left panels) or primary cardiomyocytes (right panels) were treated (or not) with IFNβ (10^5^ U/mouse or 100 U/ml respectively) and, 24 hours later, liver / heart / cells were harvested, proteins were isolated, and expression levels of IFIT2 and IFIT3 were determined by western blot.
(TIF)

S3 Fig IFIT family gene expression in cardiomyocytes is undetectable in IFIT locus KO mice, even after IFNβ treatment (related to Fig 3). Primary cardiomyocytes were isolated from B6 and IFITKO mice and treated with IFNβ (1 kU/ml) for the indicated period. Induction of the indicated IFIT family genes are shown. Each value was normalized to the values of Gapdh gene and divided by the values of uninfected controls (n = 1).
(TIF)

S4 Fig Decreased apoptotic cells and increased Iba-1-positive cells in liver of IFITKO mice after CVB3 infection (related to Figs 4 and 5). B6 and IFITKO mice were infected with CVB3 (10^4^ pfu/mouse i.p.). Immunostaining of vibratome sections of liver of the mice (12 days p.i.) were imaged by confocal microscopy. Iba-1 (Red), F-actin (Green), and nuclei (Blue).
(TIF)

S5 Fig IFIT locus is required for restricting cardiac virus replication and for preventing cardiac inflammation at 10^3^ pfu CVB3 challenge (related to Figs 4 and 6). B6 and IFITKO mice were infected with CVB3 (10^3^ pfu/mouse i.p.) and sacrificed at 12 days p.i. (A) Virus titers in the heart are represented as PFU/gram. Each symbol represents an individual value (geometric means). Asterisk indicates statistical significance (*P < 0.05). (B) Histological sections of hearts stained with Masson’s trichrome of representative mice (12 days p.i.) are shown.
(TIF)

S6 Fig The IFIT locus is required for successful IFNβ treatment of CVB3-infected mice (related to Fig 4). B6 and IFITKO mice were infected with CVB3 (10^4^ pfu/mouse, i.p.). 24 hours later, mice were treated with either PBS (open circles) or recombinant IFNβ (2 × 10^4^ units/mouse, i.p.; blue circles). The mice were sacrificed at 12 days p.i., and body weight loss and viral titers were determined in the pancreas and liver. The body weight of each individual mouse was set as 100%. In the non-treated group, the liver titer at day 12 p.i. is slightly lower than that observed in a separate experiment (see E).
(TIF)

S7 Fig IFITKO mice accelerated / stronger chemokine responses to CVB3 infection (related to Fig 5). Fold gene induction of indicated chemokines in the hearts of B6 and IFITKO mice at 1 day post-CVB3 infection (10^4^ pfu, n = 4, Means + SEM).
(TIF)

S1 Table The table shows the sequences of oligonucleotides used for PCR and CRISP/Cas9 gene editing. For PCR oligos, both the forward and reverse primers are shown. For CRISPR/Cas9 oligos, the forward and reverse sequences shown were hybridized, then cloned into the expression vector. All oligos are written in 5’ to 3’ orientation.
(DOCX)
