# Supplementary figures and images for: Biphasic and cardiomyocyte-specific IFIT activity protects cardiomyocytes from enteroviral infection
Source: PLoS Pathog. 2019 Apr 8;15(4):e1007674. doi: 10.1371/journal.ppat.1007674 (PMC6453442; doi:10.1371/journal.ppat.1007674)

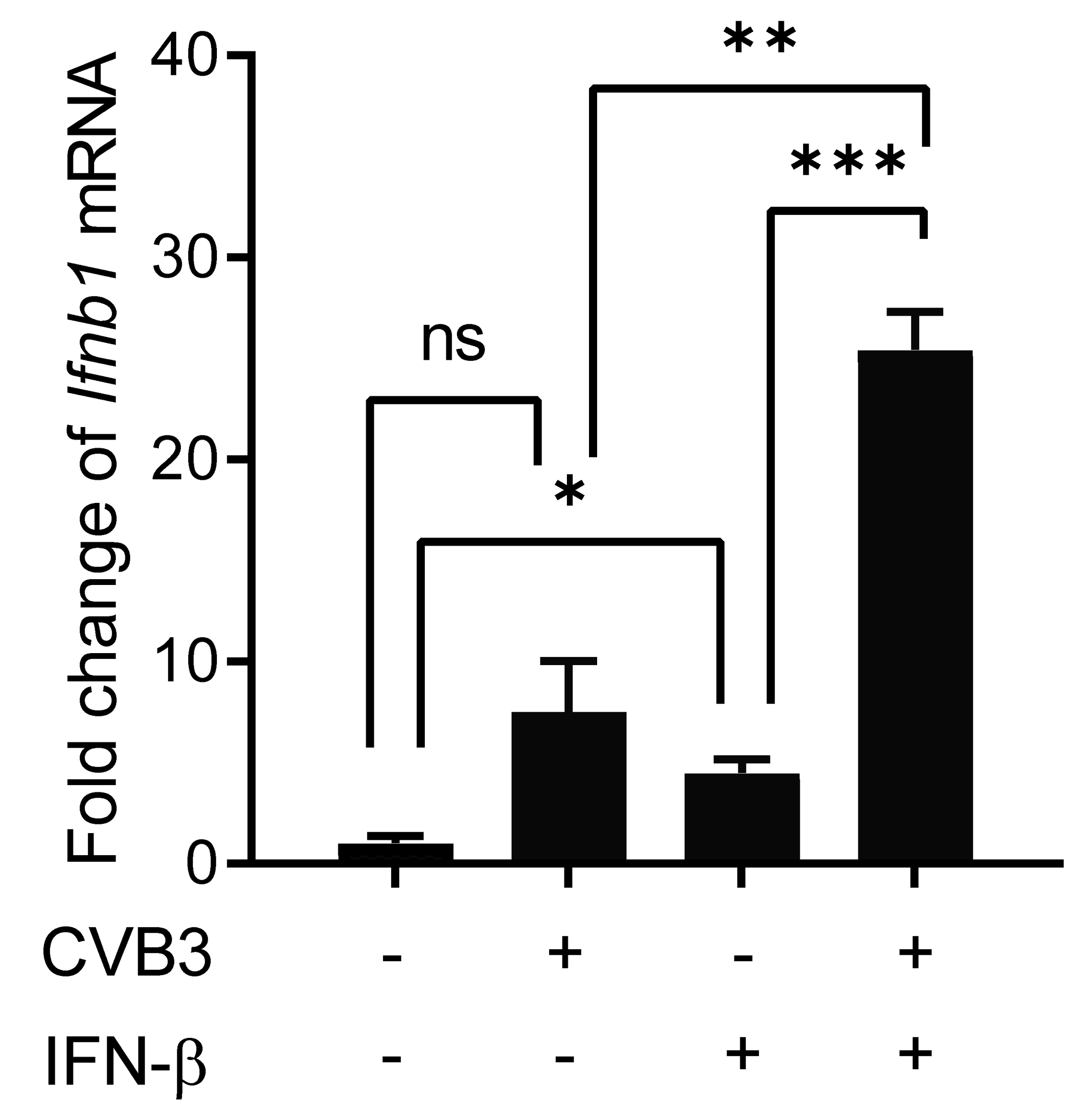

Supplement: S1 Fig — HL-1 cells were pre-treated (or not) with IFN-b for 16 hours. The cells were washed, and aliquots were infected with CVB3 at an moi of 10. 3 hours later, the cells were harvested and RNA was isolated. The abundance of IFN-b transcripts was determined using qPCR. As shown, (i) consistent with published data (see main text), CVB3 infection alone induces only a small, and statistically non-significant, increase in IFNβ in cardiomyocytes; (ii) IFNβ alone causes a statistically-significant increase in its own transcription and (iii) in IFNβ-pretreated cells, CVB3 infection now results in a dramatic (25-fold) increase in IFNβ transcript levels compared to untreated, non-infected cells. Taken together with our in vivo observations (Fig 1B & 1C), these data indicate that both in vivo and in tissue culture, the abundant transcription of IFNβ requires both T1IFN signaling and CVB3 infection. (TIF) [file ppat.1007674.s001.tif]

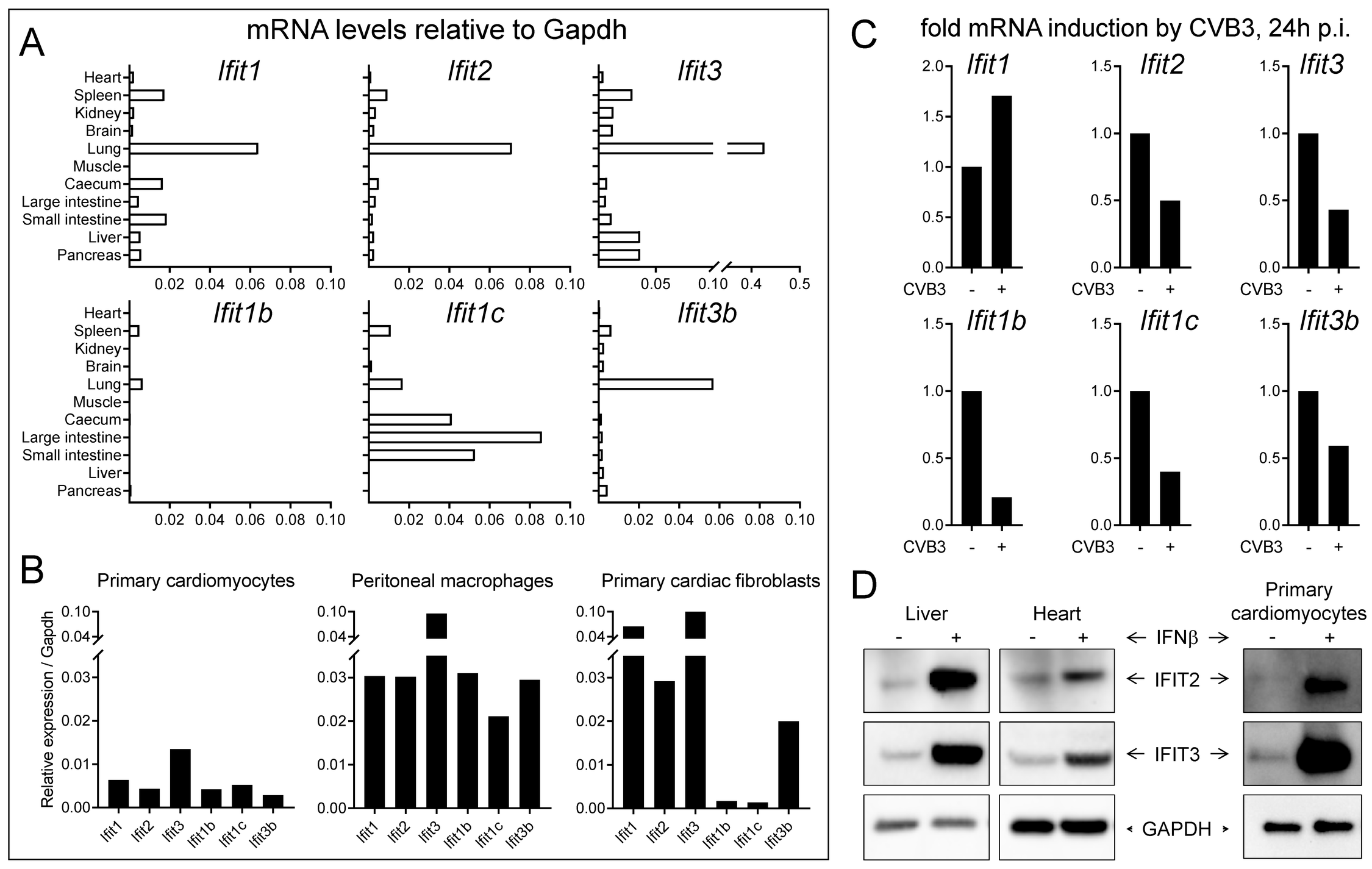

Supplement: S2 Fig — (A) Uninfected B6 mice were sacrificed and RNA was isolated from the indicated tissues. (B) Primary cardiomyocytes, peritoneal macrophages and cardiac fibroblasts were obtained from B6 mice, and RNA was isolated. For A & B, expression of mRNAs from the indicated IFIT family genes were analyzed; each value was normalized to the value for the Gapdh mRNA. (C) B6 mice were infected with CVB3 (104 pfu/mouse, i.p.) and, 24 hours later, were sacrificed. Fold changes in the expression of IFIT mRNAs (compared to uninfected hearts) were determined. (D) B6 mice (left panels) or primary cardiomyocytes (right panels) were treated (or not) with IFNβ (105 U/mouse or 100 U/ml respectively) and, 24 hours later, liver / heart / cells were harvested, proteins were isolated, and expression levels of IFIT2 and IFIT3 were determined by western blot. (TIF) [file ppat.1007674.s002.tif]

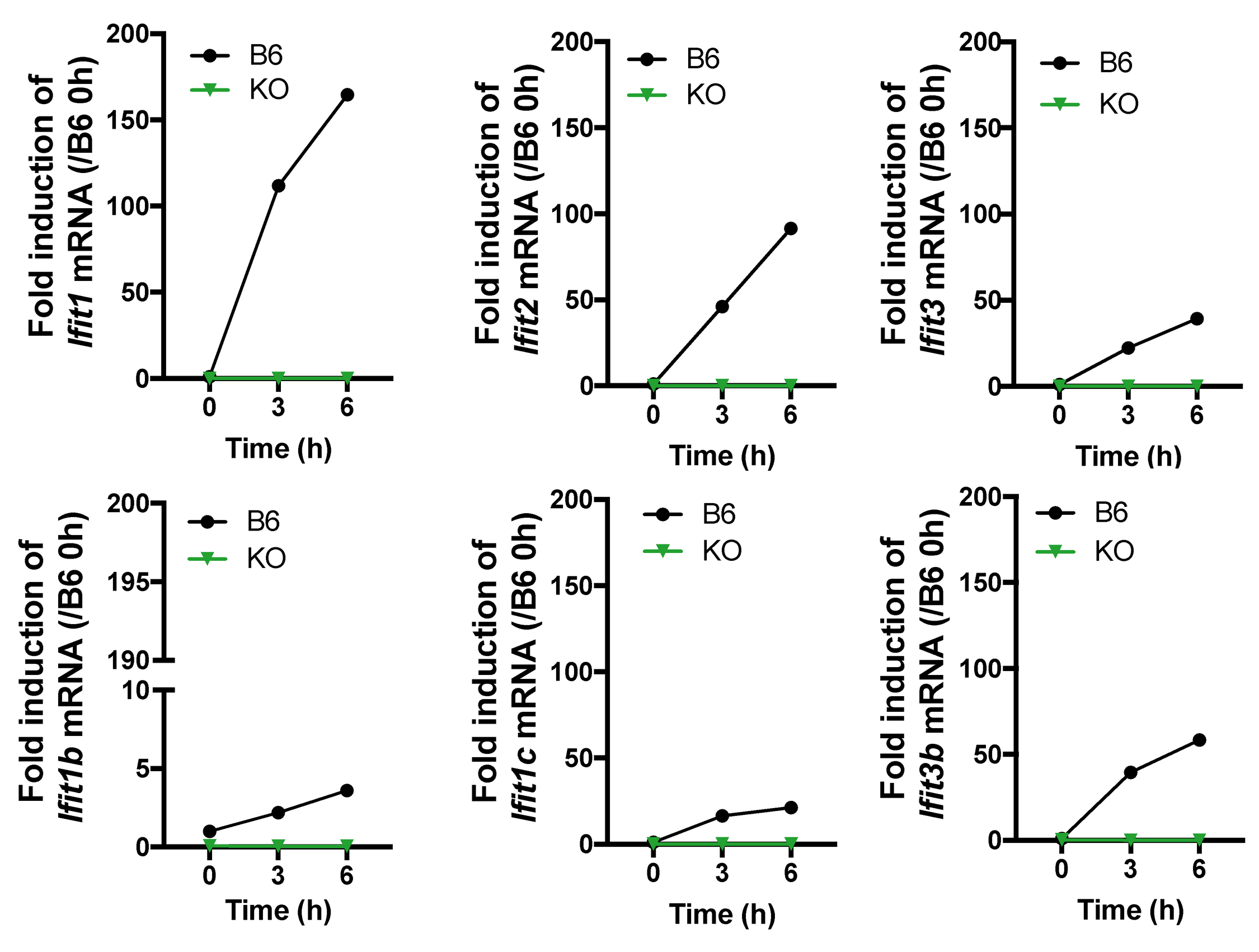

Supplement: S3 Fig — Primary cardiomyocytes were isolated from B6 and IFITKO mice and treated with IFNβ (1 kU/ml) for the indicated period. Induction of the indicated IFIT family genes are shown. Each value was normalized to the values of Gapdh gene and divided by the values of uninfected controls (n = 1). (TIF) [file ppat.1007674.s003.tif]

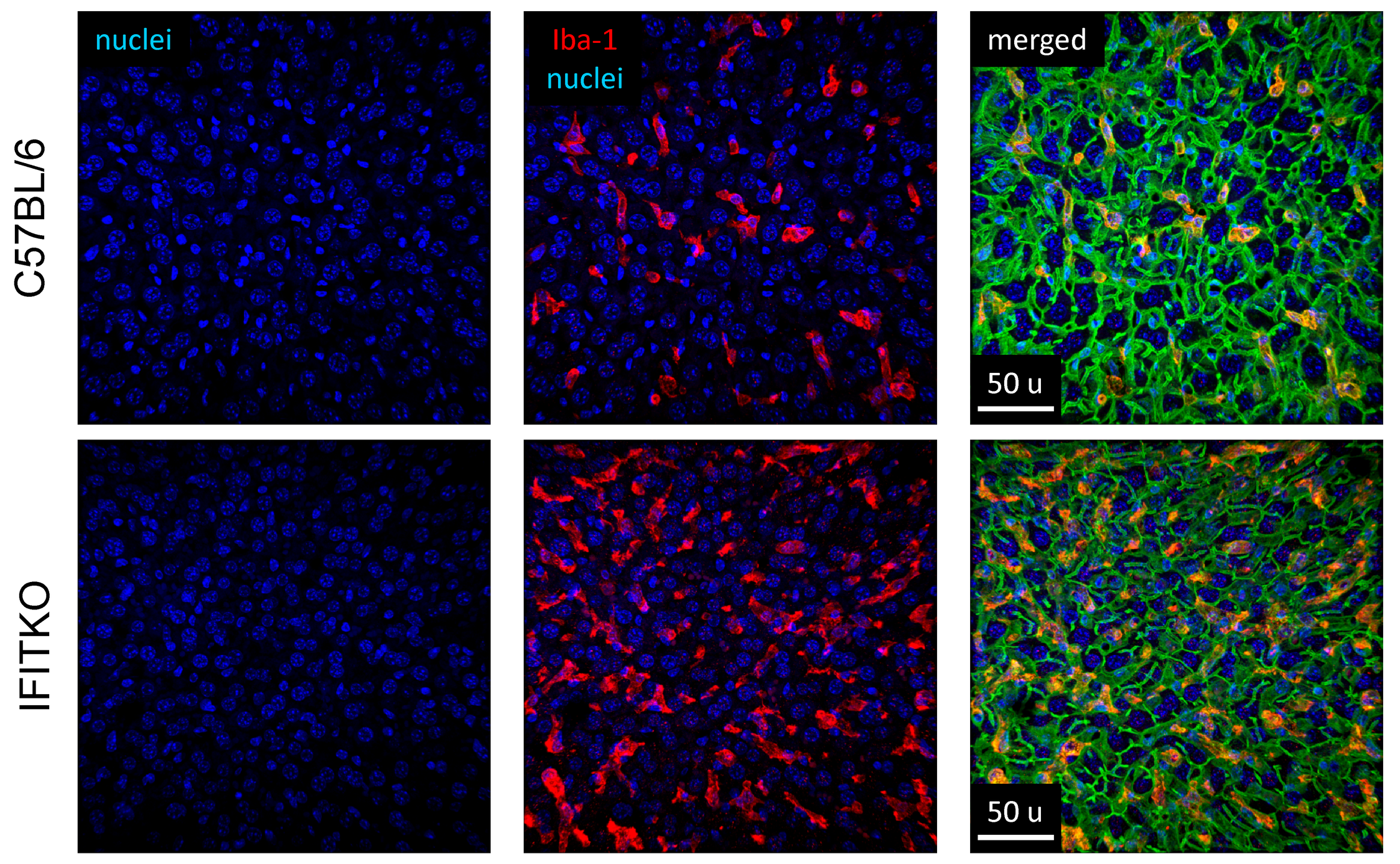

Supplement: S4 Fig — B6 and IFITKO mice were infected with CVB3 (104 pfu/mouse i.p.). Immunostaining of vibratome sections of liver of the mice (12 days p.i.) were imaged by confocal microscopy. Iba-1 (Red), F-actin (Green), and nuclei (Blue). (TIF) [file ppat.1007674.s004.tif]

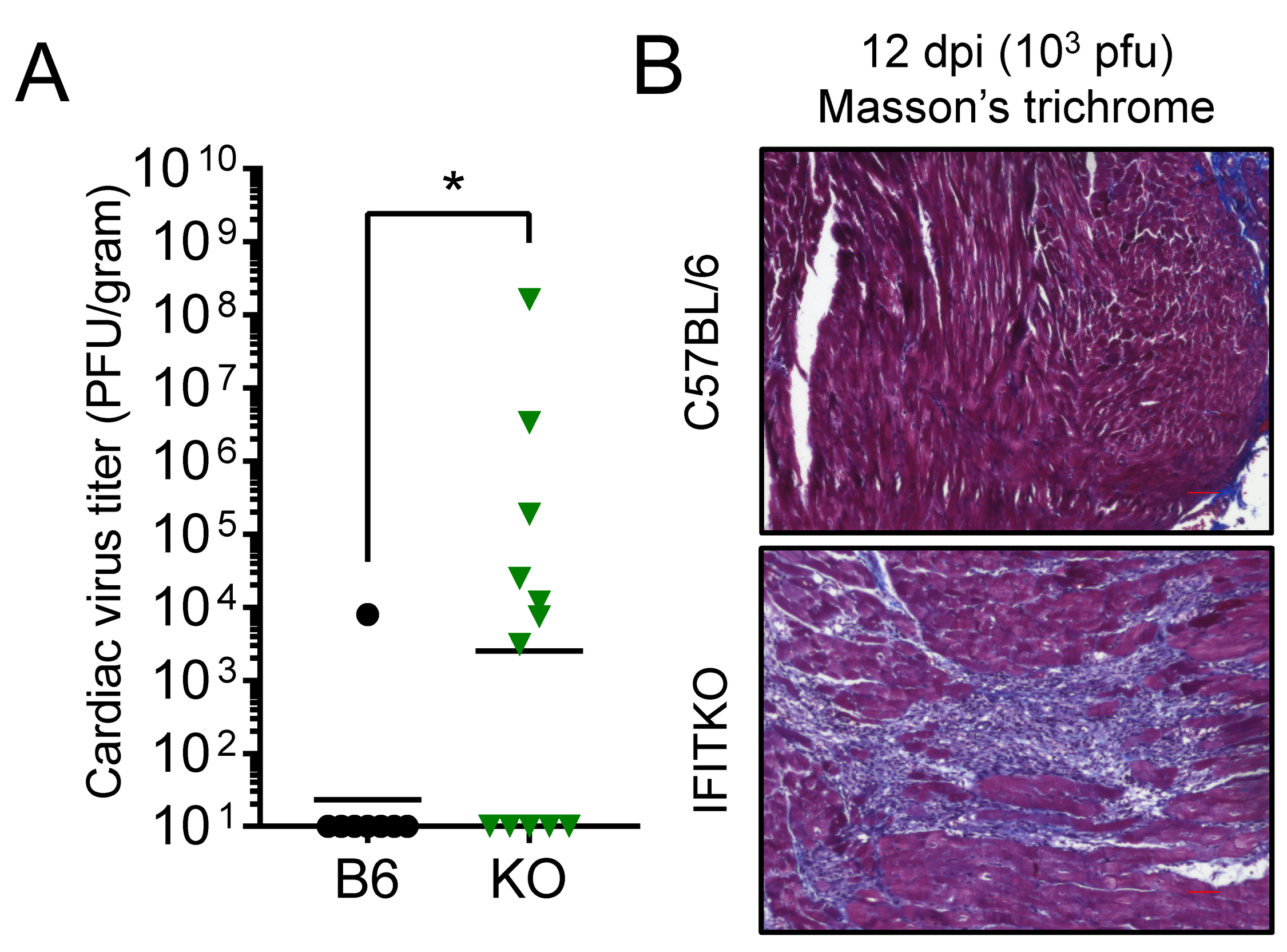

Supplement: S5 Fig — B6 and IFITKO mice were infected with CVB3 (103 pfu/mouse i.p.) and sacrificed at 12 days p.i. (A) Virus titers in the heart are represented as PFU/gram. Each symbol represents an individual value (geometric means). Asterisk indicates statistical significance (*P < 0.05). (B) Histological sections of hearts stained with Masson’s trichrome of representative mice (12 days p.i.) are shown. (TIF) [file ppat.1007674.s005.tif]

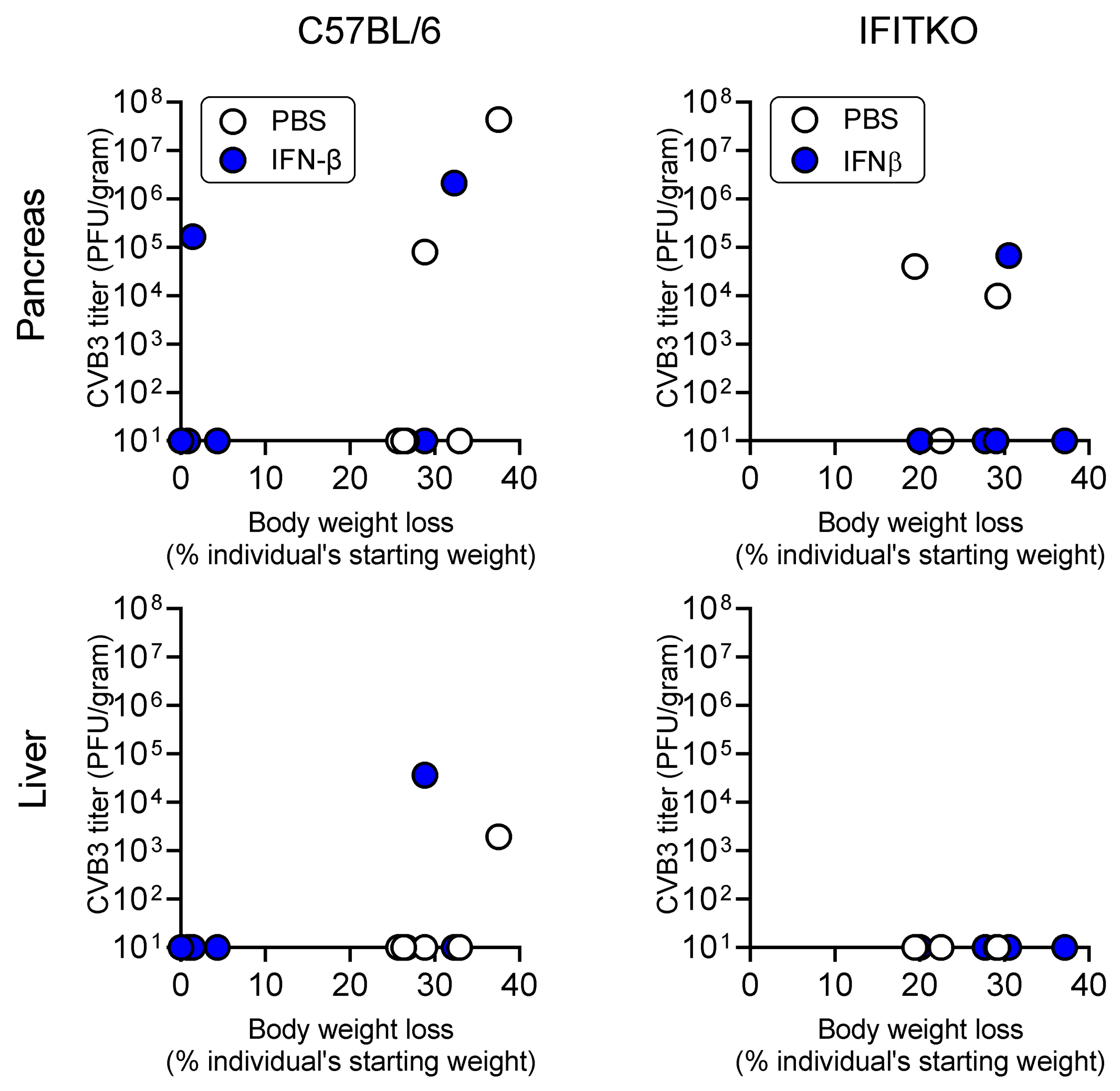

Supplement: S6 Fig — B6 and IFITKO mice were infected with CVB3 (104 pfu/mouse, i.p.). 24 hours later, mice were treated with either PBS (open circles) or recombinant IFNβ (2 × 10^4^ units/mouse, i.p.; blue circles). The mice were sacrificed at 12 days p.i., and body weight loss and viral titers were determined in the pancreas and liver. The body weight of each individual mouse was set as 100%. In the non-treated group, the liver titer at day 12 p.i. is slightly lower than that observed in a separate experiment (see E). (TIF) [file ppat.1007674.s006.tif]

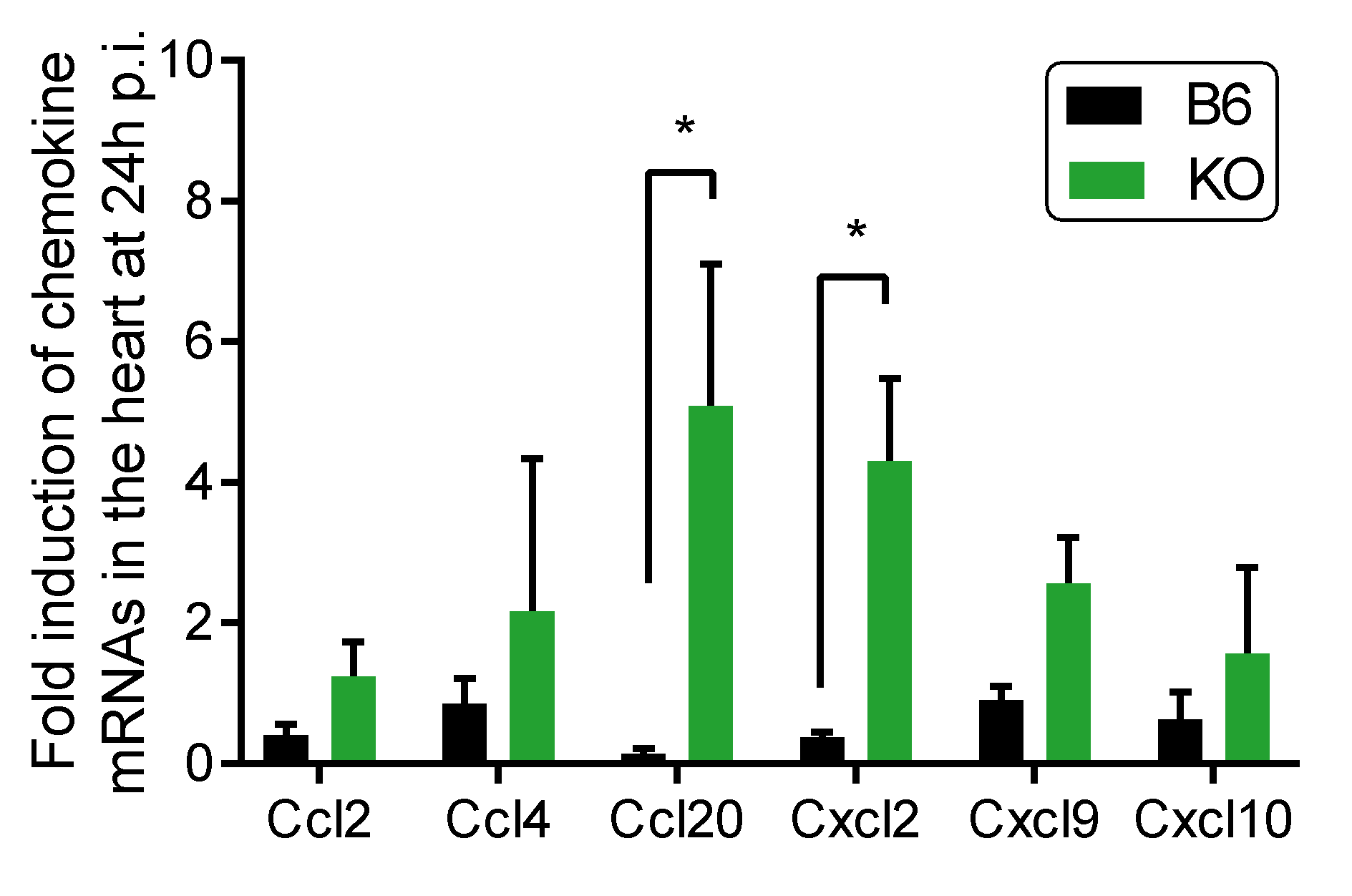

Supplement: S7 Fig — Fold gene induction of indicated chemokines in the hearts of B6 and IFITKO mice at 1 day post-CVB3 infection (104 pfu, n = 4, Means + SEM). (TIF) [file ppat.1007674.s007.tif]
