# Supplementary material for: Biphasic and cardiomyocyte-specific IFIT activity protects cardiomyocytes from enteroviral infection
Source: PLoS Pathog. 2019 Apr 8;15(4):e1007674. doi: 10.1371/journal.ppat.1007674 (PMC6453442; doi:10.1371/journal.ppat.1007674)
Supplement: S1 Table — For PCR oligos, both the forward and reverse primers are shown. For CRISPR/Cas9 oligos, the forward and reverse sequences shown were hybridized, then cloned into the expression vector. All oligos are written in 5’ to 3’ orientation. (DOCX) [file ppat.1007674.s008.docx]

**Supporting Table 1 – Oligonucleotides used for PCR and CRISPR/Cas gene editing**

| Real-time PCR for Ifit1 |
| --- |
| Forward: CTGAGATGTCACTTCACATGGAA |
| Reverse: GTGCATCCCCAATGGGTTCT |
| Real-time PCR for Ifit2 |
| Forward: AGTACAACGAGTAAGGAGTCACT |
| Reverse: AGGCCAGTATGTTGCACATGG |
| Real-time PCR for Ifit3 |
| Forward: CCTACATAAAGCACCTAGATGGC |
| Reverse: ATGTGATAGTAGATCCAGGCGT |
| Real-time PCR for Ifit1b |
| Forward: TGCCCTACTGCATTCTCCACTG |
| Reverse: GATAAGAGTCTCTGGGTGGCAGG |
| Real-time PCR for Ifit1c |
| Forward: TGCAAAATCTCATAGTTGTC |
| Reverse: GCTGGCATCAAAGAACTCA |
| Real-time PCR for Ifit3b |
| Forward: AGACAGGGTGTGCAACCAGC |
| Reverse:CGTGACCAGTCGGCGAATTTCTGCTTG |
| Real-time PCR for Cd4 |
| Forward: TCCTTCCCACTCAACTTTGC |
| Reverse: AAGCGAGACCTGGGGTATCT |
| Real-time PCR for Cd8b1 |
| Forward: TCAAGACGGCCCTTTCTCAGT |
| Reverse: ACCGTCGCGCAGAAGTAGA |
| Real-time PCR for Emr1 |
| Forward: GAGACGTTTGCCCTGAACATG |
| Reverse:AGGATCTGAAAAGTTGGCAAAGA |
| Real-time PCR for Ly6G |
| Forward: TGGACTCTCACAGAAGCAAAG |
| Reverse: GCAGAGGTCTTCCTTCCAACA |
| Real-time PCR for CVB3 RNA |
| Forward: CACACTCCGATCAACAGTCA |
| Reverse: GAACGCTTTCTCCTTCAACC |
| Real-time PCR for Gapdh |
| Forward: AGGTCGGTGTGAACGGATTTG |
| Reverse: TGTAGACCATGTAGTTGAGGTCA |
| Real-time PCR for Ccl2 |
| Forward: TTAAAAACCTGGATCGGAACCAA |
| Reverse: GCATTAGCTTCAGATTTACGGGT |
| Real-time PCR for Ccl4 |
| Forward: TTCCTGCTGTTTCTCTTACACCT |
| Reverse: CTGTCTGCCTCTTTTGGTCAG |
| Real-time PCR for Ccl20 |
| Forward: GCCTCTCGTACATACAGACGC |
| Reverse: CCAGTTCTGCTTTGGATCAGC |
| Real-time PCR for Cxcl2 |
| Forward: CCAACCACCAGGCTACAGG |
| Reverse: GCGTCACACTCAAGCTCTG |
| Real-time PCR for Cxcl9 |
| Forward: GGAGTTCGAGGAACCCTAGTG |
| Reverse: GGGATTTGTAGTGGATCGTGC |
| Real-time PCR for Cxcl10 |
| Forward: CCAAGTGCTGCCGTCATTTTC |
| Reverse: GGCTCGCAGGGATGATTTCAA |
| sgRNA sequence for Ifnar1 |
| Sense: CACCGATGACAACTACACCCTAAAG |
| Anti-sense: AAACCTTTAGGGTGTAGTTGTCATC |
| sgRNA sequence for Ifit1 |
| Sense: CACCGCAGGATATTCACCTCCGCTA |
| Anti-sense: AAACTAGCGGAGGTGAATATCCTGC |
| sgRNA sequence for Ifit2 |
| Sense: CACCGGGCCATTGCGAACTACCGTC |
| Anti-sense: AAACGACGGTAGTTCGCAATGGCCC |
| Genotyping PCR for entire IFIT family gene edited cells |
| P1: AGATGAGTCCTTGGATGAGT |
| P2: GCCAAAGCTATGTCATTCGC |
| Reverse: GGCTGGTACTCTCTACAAGA |
